# Mosaic structural variation in children with developmental disorders

**DOI:** 10.1093/hmg/ddv033

**Published:** 2015-01-29

**Authors:** Daniel A. King, Wendy D. Jones, Yanick J. Crow, Anna F. Dominiczak, Nicola A. Foster, Tom R. Gaunt, Jade Harris, Stephen W. Hellens, Tessa Homfray, Josie Innes, Elizabeth A. Jones, Shelagh Joss, Abhijit Kulkarni, Sahar Mansour, Andrew D. Morris, Michael J. Parker, David J. Porteous, Hashem A. Shihab, Blair H. Smith, Katrina Tatton-Brown, John L. Tolmie, Maciej Trzaskowski, Pradeep C. Vasudevan, Emma Wakeling, Michael Wright, Robert Plomin, Nicholas J. Timpson, Matthew E. Hurles

**Affiliations:** 1 Wellcome Trust Sanger Institute, Hinxton, Cambridge CB10 1HH, UK,; 2 Manchester Centre for Genomic Medicine, Central Manchester University Hospitals, NHS Foundation Trust, Manchester Academic Health Science Centre (MAHSC), Manchester M13 9WL, UK,; 3 College of Medical, Veterinary and Life Sciences, University of Glasgow, Glasgow G12 8QQ, UK,; 4 University Hospitals of Leicester, NHS Trust, Leicester Royal Infirmary, Leicester LE1 5WW, UK,; 5 MRC Integrative Epidemiology Unit, University of Bristol, Oakfield House, Oakfield Grove, Bristol BS8 2BN, UK,; 6 Northern Genetics Service, Newcastle upon Tyne Hospitals NHS Trust, Newcastle upon Tyne NE1 3BZ, UK,; 7 Southwest Thames Regional Genetics Centre, St George's Healthcare NHS Trust, London SW17 0RE, UK,; 8 Manchester Centre for Genomic Medicine, Institute of Human Development, Faculty of Medical and Human Sciences, University of Manchester, MAHSC, Manchester M13 9WL, UK,; 9 West of Scotland Clinical Genetics Service, Southern General Hospital, Glasgow DD1 9SY, UK,; 10 School of Molecular, Genetic and Population Health Sciences, University of Edinburgh Medical School, Teviot Place, Edinburgh EH8 9AG, UK,; 11 Sheffield Clinical Genetics Service, Sheffield Children's Hospital, Western Bank, Sheffield, UK,; 12 Medical Genetics Section, Molecular Medicine Centre, Institute of Genetics and Molecular Medicine, University of Edinburgh, Edinburgh EH4 2XU, UK,; 13 School of Medicine, Dundee University, Mackenzie Building, Kirsty Semple Way, Ninewells Hospital and Medical School, Dundee DD2 4RB, UK,; 14 King's College London, MRC Social, Genetic and Developmental Psychiatry Research Centre, Institute of Psychiatry, Psychology & Neuroscience, De Crespigny Park, London SE5 8AF, UK and; 15 North West Thames Regional Genetics Service, North West London Hospitals NHS Trust, Watford Rd, Harrow HA1 3UJ, UK

## Abstract

Delineating the genetic causes of developmental disorders is an area of active investigation. Mosaic structural abnormalities, defined as copy number or loss of heterozygosity events that are large and present in only a subset of cells, have been detected in 0.2–1.0% of children ascertained for clinical genetic testing. However, the frequency among healthy children in the community is not well characterized, which, if known, could inform better interpretation of the pathogenic burden of this mutational category in children with developmental disorders. In a case–control analysis, we compared the rate of large-scale mosaicism between 1303 children with developmental disorders and 5094 children lacking developmental disorders, using an analytical pipeline we developed, and identified a substantial enrichment in cases (odds ratio = 39.4, *P*-value 1.073e − 6). A meta-analysis that included frequency estimates among an additional 7000 children with congenital diseases yielded an even stronger statistical enrichment (*P*-value 1.784e − 11). In addition, to maximize the detection of low-clonality events in probands, we applied a trio-based mosaic detection algorithm, which detected two additional events in probands, including an individual with genome-wide suspected chimerism. In total, we detected 12 structural mosaic abnormalities among 1303 children (0.9%). Given the burden of mosaicism detected in cases, we suspected that many of the events detected in probands were pathogenic. Scrutiny of the genotypic–phenotypic relationship of each detected variant assessed that the majority of events are very likely pathogenic. This work quantifies the burden of structural mosaicism as a cause of developmental disorders.

## Introduction

Developmental disorders (DD) are diseases of impaired prenatal development and arise from several genetic mechanisms. The most common mutational category reported in children with DD is *de novo* mutations (1,2). *De novo* mutations that occur post-zygotically result in genetically heterogeneous cellular populations, a phenomenon known as *mosaicism*. As mutations arise with every cell division, strictly speaking, all humans are mosaic. Nevertheless, physiology-disruption (pathogenic) mosaicism is more likely to occur from high-frequency mutations, and here we focus on abnormalities with sufficient cellular frequency to be detected by current microarray technology. Another cause of genetically distinct cell populations, although much rarer, is chimerism, owing to the fusion of cell lineages from different zygotes (3). The detection sensitivity of mosaic abnormalities is a function of several parameters, some of which are intrinsic to the mosaic event—including event size, clonality, type (loss, gain, and LOH); others which are technology dependent—including platform (karyotyping or microarray), company, number of probes, signal-to-noise ratio of probes; and others which are algorithmic—such as single-sample versus trio-based tests. In this study, we primarily focus on large mosaic abnormalities in at least ∼10% of cells using single-nucleotide polymorphism (SNP) microarray and single-sample tests.

Mosaicism can involve multicellular clonality for mutations of any size (3,4). While the detection of mosaic point mutations has been used for the validation of suspected mosaicism at specific genomic positions in rare disease and cancer (5,6), reliable detection of small-scale mosaicism genome wide requires very high-depth whole-genome sequencing, which is not currently economical for widespread clinical application. At the other end of the size continuum, cytogenetic karyotyping has been used for decades in a clinical diagnostic setting to detect microscopically visible (5–10 Mb or larger) abnormalities, including mosaic events, in children with congenital disorders. While karyotyping is still widely used in many centres, this approach is insensitive to sub-microscopic rearrangements and supernumerary marker chromosomes (7) and is labour-intensive, because, for example, 30 cells must be counted to exclude 10% mosaicism with 95% confidence (8). Compared with karyotyping, SNP genotyping chips offer a higher-resolution, higher-throughput assay and are considered a standard of care for clinical diagnostics in children with developmental disabilities (9). The resolution of SNP chips for mosaicism detection is influenced by probe density and the signal-to-noise ratio of the experiment and the type of mosaic abnormality. In this study, we focussed on mosaic events of at least 2 Mb in size, a generally accepted threshold for large structural alterations (10), allowing a fair basis of comparison for the different chip designs we analysed, and concordant with a recent study that used an SNP chip design and algorithmic protocol similar to our own (11). Henceforth, the term *mosaicism* will refer to mosaic events of at least 2 Mb in size.

The SNP platform generates a measure of allelic intensity, the log R ratio (LRR) and a measure of allele balance, the B-allele frequency (BAF). When genetic heterogeneity exists in assayed cell populations, the BAF will be skewed from expected diploid frequencies, and software tools translate deviation of BAF into mosaic detections. Mosaic Alteration Detection (12) (MAD), is a popular software tool that detects deviations in BAF, groups nearby segments into clusters and uses a statistical test to determine clusters that are statistically unlikely at a given significance threshold. Once such clusters are selected, the average LRR value in each segment is used to classify segments into mosaic type: loss, gain or loss of heterozygosity (LOH). The detection sensitivity for MAD on SNP chips with ∼1 million probes for events at least 2 Mb in size is limited to loss or LOH events in ∼10–90% of cells and gain events in ∼20–80% of cells (11,12). Detection power can be improved if phased genotype data are available, as it can then be shown that adjacent deviations in BAF arise from the same haplotype, which is less likely by chance alone. triPOD (13) is a trio-based mosaic detection tool that leverages parental genotype data to phase child genotypes and has been shown to have increased sensitivity, compared with MAD, for detecting events below ∼10% clonality, but this software tool requires parent genotype data, which are not always available.

MAD was recently implemented on ∼60 000 adults and identified a strong positive correlation between the age of the sampled individuals and mosaicism frequency (11). Several studies have measured mosaicism frequency among children ascertained for clinical diagnostic testing (Table 1) and have derived estimates from ∼0.2–1%. In comparison with studies of clinically ascertained children with DD, the prevalence of mosaicism among children without DD is less well established, although evidence suggests that the frequency is extremely low (11,19). In the cohort studies analysed by Laurie, no mosaicism was detected in any of 1600 individuals aged 10–19 years. While 13 mosaic events were found among 6810 children aged 0–4, a frequency of 0.19%, this may reflect ascertainment bias, as the youngest stratum of children in this study included children from a cohort study of oral clefts, a potential manifestation of pathogenic mosaicism. Thus, the frequency of mosaicism in children without DD remains an open question.

**Table 1.** Clinical diagnostic microarray studies of children with congenital or developmental abnormalities

	Platform	No. of probes	Tissue	No. samples	No. mosaics	Frequency (%)
Bruno	Illumina HumanCytoSNP-12	220 k	Blood, skin biopsy and saliva	5000	13	0.26
Conlin	IlluminaQuad610 (SNP)	620 k	Blood and fibroblasts	2019	23 (1 chimera)	1.1
Ballif	SignatureChip CGH	969 BACs	Blood	3600	18	0.5
Cheung	CGH	853 BACs	Blood	2585	18	0.5
Pham	BCM V8 OLIGO (aCGH)	180 k	Blood	10 362	57	0.55

SNP, single-nucleotide polymorphism; aCGH, array comparative genomic hybridization; BACs, bacterial artificial chromosomes; (14–18).

In this study, to quantify the burden of pathogenic structural mosaicism in children with DDs, we determined the frequency of structural mosaicism in thousands of children with and without DD, using both single-sample (MAD) and trio-based (triPOD) detection of structural mosaicism from SNP chip data. Both clinical review of the specific variants and a statistical analysis of enrichment of structural mosaicism in cases indicated that the majority of the mosaic events detected in probands were pathogenic.

## Results

To estimate the frequency of structural mosaicism in children with and without DD, we compiled SNP genotyping data on DNA from blood or saliva from three studies: a trio-based study of children with DD, the Deciphering Developmental Disorders (DDD) study (*N* = 1303) (20); two UK birth cohort studies: the Avon Longitudinal Study of Parents and Children (ALSPAC, *N* = 2168) (21) and the Twins Early Development Study (TEDS, *N* = 3588) (22). In case–control analyses based on single-sample detection of structural mosaicism (using the MAD algorithm), we compared DDD cases with a control set that included ALSPAC and TEDS children lacking delayed development.

Additionally, we implemented trio-based detection of structural mosaicism (using the triPOD algorithm), using two studies with trio data available: DDD, and the Scottish Family Health Study, a study of young-adult healthy controls and their parents [Scottish Family Health Service (SFHS), *N* = 478] (23).

**Figure 1.**
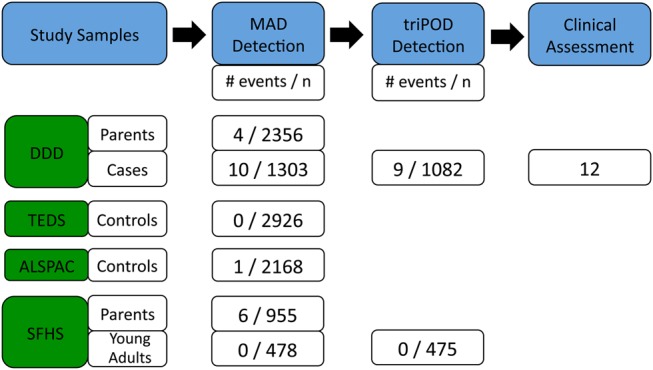
Overview. A MAD-based workflow was used to detect mosaicism. This workflow identified an enrichment of mosaicism in cases compared with controls, and triPOD detected two additional mosaic events not detected by MAD. Clinical assessment was performed on all 12 probands of the DDD study with mosaicism.

Below we describe the pipelines we developed to detect and filter candidate mosaic events, and then we characterize the mosaic events detected in probands and their likely clinical significance (Fig. 1).

### Mosaic detection workflow

Initial testing of MAD on DDD study data produced >1000, mostly spurious, putative structural mosaic events. The predominant recurrent source of these erroneous signals was due to incorrect classification of long tracts of constitutive homozygosity as being mosaic; such homozygous tracts are relatively frequently observed in the DDD study as families often have familial relatedness (24), which results in large blocks of inherited homozygosity (identity by descent). Moreover, excess putative detections frequently arose from over-segmentation of single contiguous regions, an artefact of imperfect delineation of event boundaries that is a common pitfall for segmentation algorithms. A smaller number of putative mosaic detections arose from misclassification of constitutive copy number events, mainly duplications, as being mosaic. These CNVs had extreme B-allele deviations and LRR values that clustered with inherited duplications and not with other *de novo* mosaic events, supporting the classification of these events as constitutive (Supplementary Material, Fig. S1, Supplementary Material, DDD & SFHS Constitutive CNV Filtering).

Automatic filtering (Materials and Methods) based on the common error modes described earlier reduced the number of putative detections by ∼90%, to a manageable number that could be manually reviewed. Manual curation was then used to filter putative detections resulting from stochastic fluctuations in the data. To avoid unintentional exclusion of mosaicism, samples were not automatically excluded on the basis of aberrant average standard deviation of heterozygous B-allele frequencies, a commonly employed QC criterion in GWAS studies; eight samples with consistent multi-band skew of BAFs across all chromosomes, a signature of contamination, were removed from analysis (Supplementary Material, Fig. S1). However, this strategy also retained one sample with a high BAF standard deviation of 0.06 and which reflected a real mosaic structural event.

**Figure 2.**
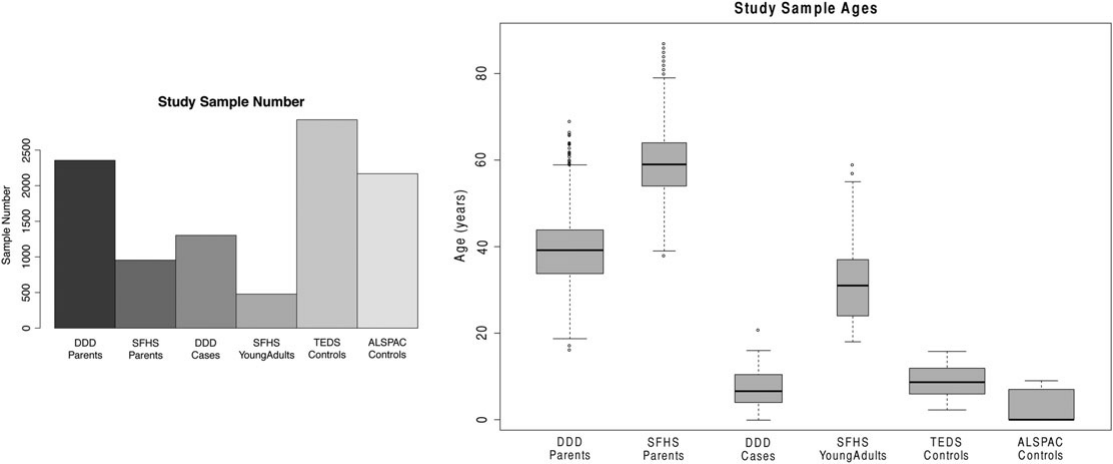
The (**A**) sample number and (**B**) ages corresponding to the analysed studies.

**Figure 3.**
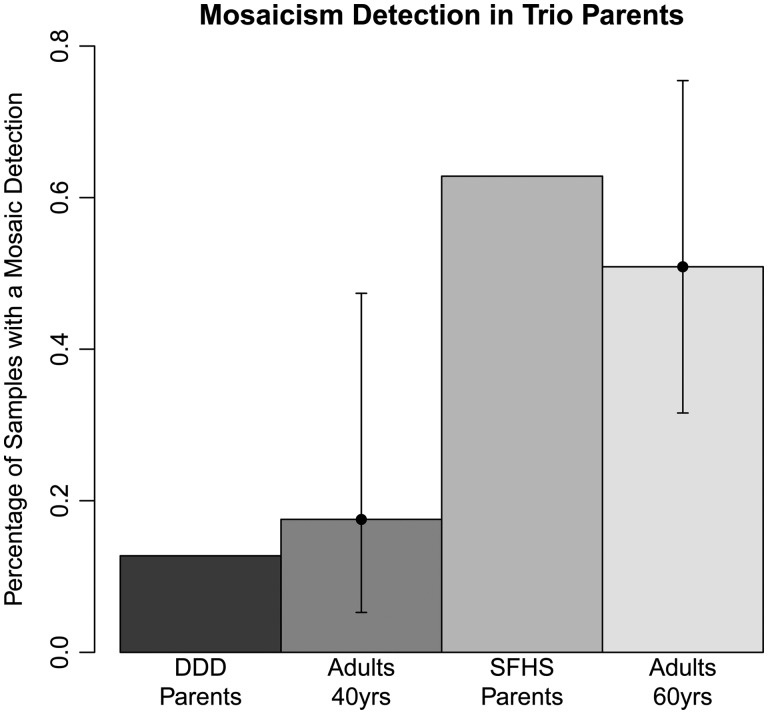
The frequency of mosaicism detected in the parents of the trio cohorts was within the confidence intervals of the frequency detected for samples of this age range.

To assess the accuracy of this MAD-based workflow, we compared the frequency of mosaic events detected among the parents of the DDD and SFHS trio studies with established estimates of mosaicism frequency for individuals of these ages. The median age at sampling of DDD parents was 39 and that of SFHS parents was 59 (Fig. 2). We identified 6 mosaic events among 955 parents of SHFS controls, a frequency of 0.6%, and 4 among 2356 parents of DDD probands, a frequency of 0.1%, which are within the confidence interval estimates for these ages (11) (Fig. 3). This suggested that the method, filtering strategy and manual curation used were not inconsistent with expectations, and we next used this workflow to detect mosaicism in the child samples.

### Estimates of mosaicism frequency in cases and controls

**Figure 4.**
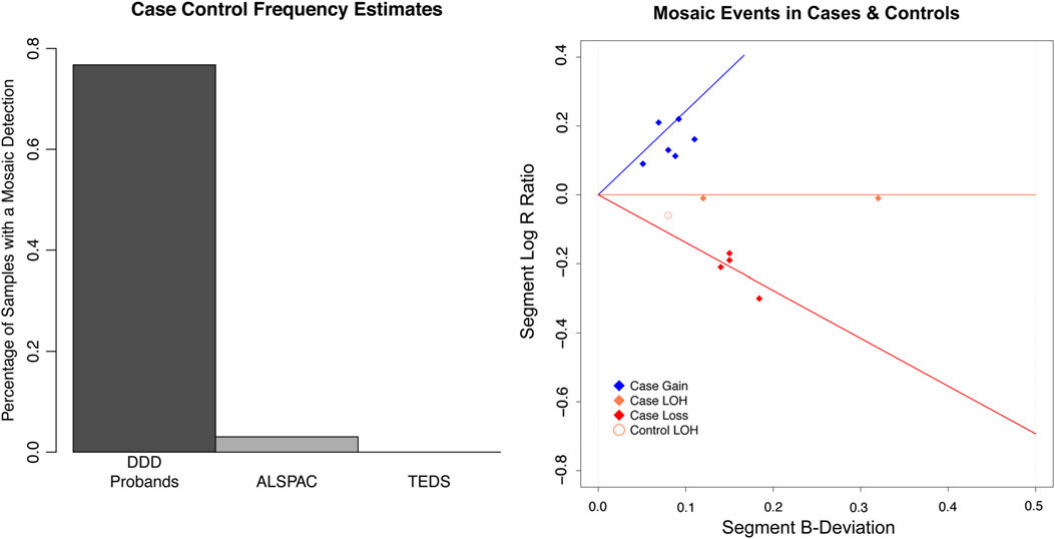
(**A**) The percentage of samples with mosaic events in the case and control cohorts. (**B**) A depiction of each mosaic event, where the line segments represent the ideal location of mosaicism for gains (blue), LOH (orange) and losses (red).

**Figure 5.**
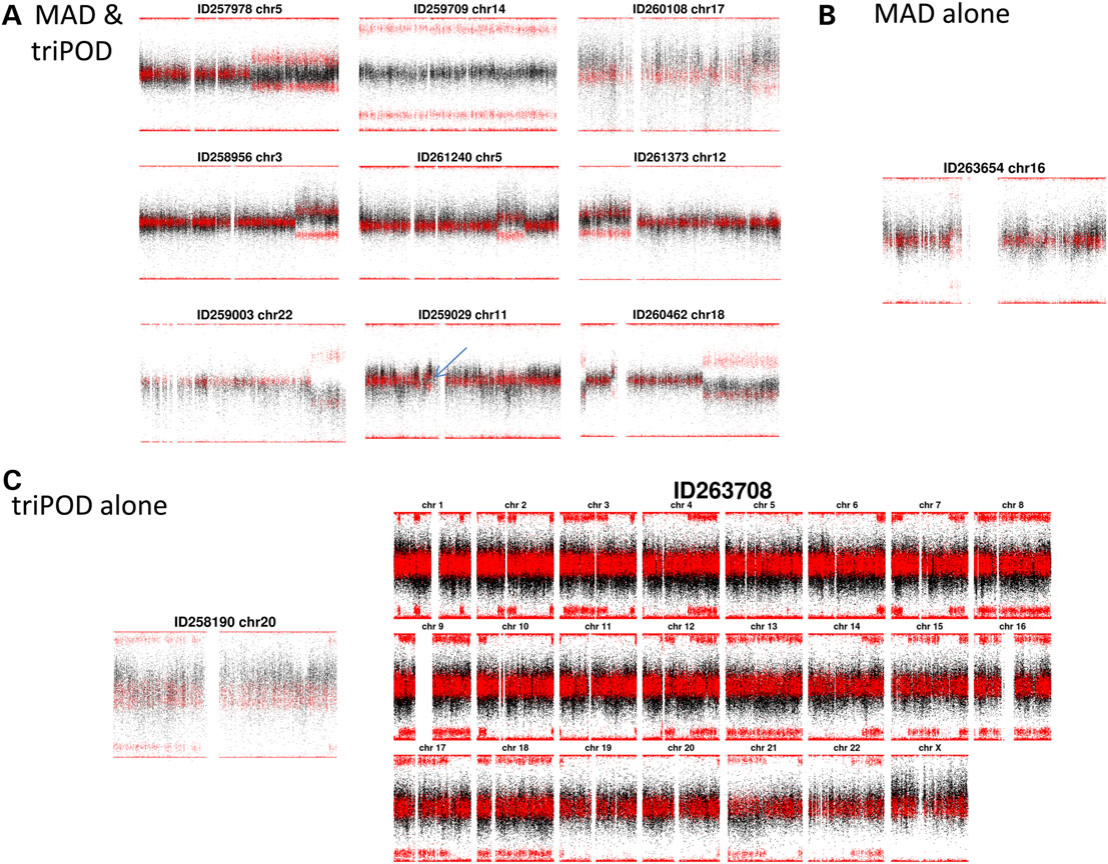
All Proband Detections: The detections made by (**A**) MAD & triPOD, (**B**) by MAD alone and (**C**) by triPOD alone.

We assessed the frequency of mosaic events in the DDD cases, and in the controls of TEDS, ALSPAC using SNP chip data. Among 1303 children from the DDD study, there were 10 with mosaic events, a rate of 0.77% (Figs 4 and 5). Compared with the estimate of mosaicism detected among children ascertained for genetic testing in Conlin *et al.*, the frequency of mosaicism in DDD was not significantly different (Fisher exact test two-sided, *P*-value 0.4698). The range of cellular fraction (clonality) was 23–66%. We investigated the distribution in saliva and blood of the eight mosaic copy number events with validation data available, finding that two were present in both saliva and blood, six were present in saliva but not blood and no events were present in blood alone.

There were 3588 children in the TEDS cohort with genotype data from blood DNA available. Analysis was performed on 2926 samples for which phenotypic data were available and samples were not medically excluded nor had developmental problems. There were zero mosaic events retained after accounting for seven constitutive detections (Supplementary Material, Computational Filtration). There were 8970 children in ALSPAC with genotype data available from DNA derived from blood or cell lines. An initial attempt at detecting mosaicism in data from both DNA sources detected more mosaicism in samples derived from cell lines (two-sided Fisher exact test *P*-value 5e − 05), suggesting the presence of cell-line-induced chromosomal rearrangements (25,26), which would overestimate *in vivo* mosaicism. To assess frequency in children accurately, we analysed the 3290 DNA samples sourced from only blood or saliva (but not cell lines). Of 2538 children with phenotypic data available, 2168 (85%) lacked DD or major developmental problems. One sample contained a mosaic LOH, representing a frequency of 0.05%.

We also investigated a collection of 478 individuals from the SFHS. These were samples without DD recruited in early adulthood, median age 31. There were zero mosaic events remaining after automated filtering and manual curation of 28 possible mosaic events.

Compared with the fraction of mosaic detections among all child control samples (2 in 5345), the frequency of mosaicism in DDD probands (10 in 1303) was highly statistically significant (odds ratio 20.66, one-sided Fisher exact test, *P*-value 3.627e − 6). A meta-analysis additionally incorporating 7119 samples from two previous studies (15,17) strongly supports a statistical enrichment of mosaicism in children with DD (*P*–value, 9.919e − 11). Before assessing the pathogenicity of these mosaic detections, we first attempted to detect additional lower-clonality mosaic events using a trio-based detection tool.

### Additional detections using triPOD

A recent trio-based algorithm, triPOD, has been shown to have improved sensitivity to detect lower-clonality mosaic events compared with MAD (13) by leveraging haplotype information in trio data. We implemented this tool on DDD trio data to improve detection of mosaic events of lower clonality. Complete trio genotypes were available for 1082 of 1303 (83%) probands, and these were processed with triPOD. There were a vast number (4920) of putative detections, of which 148 were at least 5 Mb and 876 were at least 2 Mb. We manually reviewed all events at least 5 Mb and analysed the subset of those at least 2 Mb with a non-zero median BAF deviation (see Materials and Methods). Detection at the 2-Mb level identified 7 of the 10 mosaic events that had been detected in single-sample analysis by MAD. Two of the three remaining events lacked complete trio data so they could not be analysed by triPOD. The third remaining undetected event was a mosaic duplication characterized by an additional haplotype not present in the diploid cell line (Fig. 5; Supplementary Material, Fig. S2).

An additional two events were identified among the 148 putative events of >5 Mb detected by triPOD that were each reviewed manually (Fig. 5). One event appeared to have a chromosome-wide elevation of LRR and a BAF pattern reflecting meiotic crossover, perhaps resulting from incomplete trisomy rescue. The second event was extraordinary for a genome-wide pattern of large segments of consistently aberrant BAF interspersed with segments of normal BAF. These segments of aberrant BAF were present on most chromosomes in three or fewer large segments per chromosome. We investigated the parental origin of these aberrant BAF segments by plotting the proband BAFs within these segments separately for each configuration of parental genotypes (Supplementary Material, Fig. S3). The sites with aberrant BAF were only observed where the father was heterozygous, suggesting that the aberrant BAF was due to the presence of both paternal chromosomes. In addition, the BAF at obligate heterozygous sites in the proband (parents homozygous for different alleles) was always skewed towards a greater contribution from the inherited paternal allele, suggesting a second paternal haplotype, and only one maternal haplotype. These observations are potentially compatible with a triploid cell line; however, karyotypic analysis failed to identify any triploid cells. An alternative explanation is ‘androgenetic/bipaternal mosaicism or chimerism’ (27,28), which occurs with fusion of a fertilized embryo and a polar body fertilized by a second sperm that undergoes endoreduplication. The homozygous BAF skews had BAF deviations consistent with approximately 15% clonality, which is a smaller cellular burden than any event detected by MAD.

triPOD was also applied to detect structural mosaicism in the 475 SFHS control trios, but zero mosaic detections were uncovered following computational filtering and manual curation.

### Clinical interpretation of probands with mosaicism

Mosaicism was detected in twelve individuals with DD (Table 2). For each proband, we collected data for the perinatal period, assessed developmental milestones and recorded phenotypes at the time of recruitment using a standardized nomenclature called the Human Phenotype Ontology (29). We assessed whether each mosaic event overlapped with regions implicated in specific genomic disorders and, if so, assessed the concordance of patient phenotypes with the manifestations of these genomic syndromes. To identify a relationship between the mosaic copy number events found in probands to CNVs listed in public databases required the assumptions that: (1) pathogenicity is due to disruption of overlapped regions, not owing to disruption of boundary elements for which the compared CNVs may differ, and (2) constitutive CNVs that are pathogenic produce phenotypes which are similar in character, if perhaps larger in magnitude than the corresponding CNV in mosaic state. We presumed that mosaic LOH mutations might result in imprinting syndromes, by disrupting differentially methylated regions (30) or manifest recessive diseases, by converting a single inherited deleterious allele to homozygosity. To investigate these possibilities, we used the proband BAF and parental genotypes to determine which proband allele was present in a homozygous state in the tissue containing the mosaic abnormality.

**Table 2.** Mosaic events detected among 1303 DDD probands

Sample	Sex	Birth records			Measurements at time of recruitment					Mosaic abnormality							Validation				
		Gestation (weeks)	Birth weight (kg)	Required NICU (days)	Age	Height (cm)	Weight (kg)	OFC (cm)	ID	Type	Chr.	Start (GRCh37)	End (GRCh37)	Size (Mb)	B-Dev	Clonality	aCGH results		FISH results		Tissue-limited?
																	Blood	Saliva	Blood	Saliva	
260462	F	37	2.6 (35)	No	5 year	89 (3)	10.86 (1)	45.5 (1)	GDD	Loss	18	6 50 816	2 804 129	2.2	0.14	0.44	No deviation	Downwards	Not detected	56% (buccal)	Yes
										Gain	18	13 422 042	15 265 500	1.8	0.1	0.5					
										Loss	18	48 362 664	78 015 180	29.7	0.1	0.46					
261240	F	37	1.9 (25)	7	16 year	152 (7)	52 (48)	53 (7)	Moderate	Gain	5	123 828 524	145 717 285	21.9	0.08	0.38	Not done	Upwards	Double ring	Not done	No
258956	F	38	2.6 (17)	10	4 week	73.5 (26)	7.58 (1)	43.8 (1)	Moderate	Gain	3	153 567 441	197 148 984	43.6	0.11	0.56	No deviation	Upwards	Failed QC	Not done	Yes
261373	F	38	2.0 (1)	No	4 year	96 (7)	14 (10)	50 (17)	Moderate	Gain	12	1 93 818	38 453 531	38.3	0.09	0.44	No deviation	Upwards	Not done	12% tetrasomy (buccal)	Yes
263654	M	32	2.2 (90)	19	7 year	100 (14)	14 (6)	47 (1)	GDD	Gain	16	27 183 151	31 888 684	4.7	0.07	0.33	No deviation	Not done	Not detected	50% (buccal)	Yes
259003	M	40	4.6 (98)	No	3 year	NA	15 (59)	51 (33)	GDD	Loss	22	47 182 944	51 666 786	4.5	0.184	0.54	Downwards	Downwards	43%	Failed QC	No
260108	F	40	3.6 (80)	?	19 week	60 (1)	5.1 (1)	38 (1)	GDD	Gain	17	66 922 993	81 006 629	14.1	0.092	0.451	No deviation	Upwards	Failed QC	Failed QC	Yes
263708	F	38	2.8 (27)	Yes, ? days	16 year	157 (14)	59 (67)	56 (75)	Moderate	GWpUPD	All	N/A	N/A	N/A	0.0477	0.174	No deviation	No deviation	Not detected	Results pending	NA
258190	M	38	5.9 (99)	7	6 year	113 (7)	22.8 (60)	55 (cm)	GDD	Gain	20	1	63 025 520	63	0.0578	0.261	No deviation	Not done	Not detected	30% (buccal)	Yes
259709	M	34	2.9 (98)	31	10 year	132 (64)	28 (67)	?	Moderate	LOH	14	20 432 664	107 287 663	86.9	0.33	0.66	No deviation	Not done	N/A	N/A	NA
257978	F	40	4.2 (95)	No	15 year	?	?	50 (4)	Severe	LOH	5	101 118 483	180 710 763	79.6	0.12	0.24	No deviation	Not done	N/A	N/A	NA
259029	F	40	3.3 (41)	No	5 year	109 (77)	18 (60)	50 (11)	Moderate	Gain	11	42 322 518	45 512 054	3.2	0.051	0.227	Pending	No deviation	Results pending	Results pending	?

NICU, Neonatal Intensive Care Unit; GWpUPD, Genome-wide paternal Uniparental Disomy; LOH, loss of heterozygosity; ID, Intellectual Disability; GDD, Global Developmental Delay; OFC, Occipital Frontal (head) Circumference.

Patient 260462 had global developmental delay, intermittent horizontal nystagmus with alternating abnormal head position and bilateral, symmetric large optic nerves. Magnetic resonance imaging of the brain showed cortical atrophy, generalized delay in myelination, moderate-sized left middle cranial fossa, arachnoid cyst and deficiency of the rostrum of corpus callosum and atrophic splenium. Copy number analysis by karyotype and aCGH, genetic testing for Pitt–Hopkins, Fragile X syndrome, *MECP2* gene test, spinal muscular atrophy and Angelman syndrome were all normal. Upon recruitment to the DDD study, aCGH was performed on blood and saliva, and no large (>500 kb) CNVs were reported. Our mosaic analysis on SNP data from a salivary sample identified three mosaic events on chromosome 18, two deletions and one duplication in ∼50% of cells. Results from triPOD showed that the deletions resulted from loss of the maternal allele, while the duplication was of the paternal allele (Supplementary Material, Fig. S4). Fluorescent *in situ* hybridization (FISH) analysis on cells from a buccal sample confirmed these events in 56 of 100 inspected cells. Retrospective scrutiny of the salivary CGH array identified deviations in aCGH probes but insufficient to be detected by the standard copy number detection pipeline. No deviation in blood aCGH probes was noted, suggesting the mosaicism was not present in all tissue types, and providing a likely explanation as why genetic testing, performed on blood, was negative. The mosaic deletion on chromosome 18 contains the gene *TCF4*, mutations in which cause Pitt–Hopkins syndrome (31), a diagnosis previously considered in this child, and the diagnosis was conveyed to the family.

Female patient 261240 required 7 days in neonatal intensive care and 2 weeks with nasogastric feeding. She had developmental delay, seizures and short stature (154 cm, third centile at 16 years). Before enrolment into DDD, clinical karyotyping was performed on blood and showed a marker chromosome originating from chromosome 5, local inspection by aCGH did not detect any CNVs and the marker chromosome was classified as a balanced rearrangement. Clinical testing for Fragile X syndrome was normal. Our mosaicism analysis was performed on a saliva sample and identified a 22-Mb duplication, present in ∼40% of assayed salivary cells. Review of the interphase karyotypic data noted that the suspected marker chromosome contained a double-ring chromosome. Retrospective manual review of the array CGH data on saliva identified stretches of raised LRR probes. Therefore, this event was classified as present in both blood and saliva. Duplications in this region, 5q23.2 to 5q32, have been previously implicated in seizure disorders [p.252] (32) and shared phenotypes and short stature are seen in a different patient with an overlapping duplication in the Decipher database (255372). Therefore, this mosaic aberration was considered very likely pathogenic.

Female patient 258956 had a number of congenital abnormalities, including a sacral meningocele, polydactyly, bilateral talipes, atrial and ventricular septal defects, pulmonary stenosis, EEG epileptiform activity, facial asymmetry, hirsuitism and hypomelanosis of Ito. At birth, she required neonatal intensive care for apnoea and nasogastric feeding for 10 days. Clinical aCGH (Agilent 8 × 60 K oligoarray) testing performed on blood was normal. The DDD aCGH results from blood and saliva showed upward deviation in the data from assayed saliva tissue, only. Our mosaicism analysis on saliva identified a 44-Mb duplication on chromosome 3q in ∼55% of assayed cells. Thus, it is likely that this event is tissue-limited. Duplications of 3q are associated with joint contractures, talipes, feeding difficulties, hirsuitism and heart defects, including ASD and VSD (33). There are several patients also present in the DECIPHER database who have duplications overlapping this large duplication in the child, including 280 551, with hirsuitism, feeding difficulties and global developmental delay; 283 584, with sacral dimple and low set ears; 1561, with frontal bossing and sacral dimple. Several examples of duplications of 3q have meningocele [p.145] (32). Given the consistency of phenotypes with the proband and these patients, the mosaic mutation was considered very likely pathogenic.

Female patient 261373 had intrauterine growth retardation with a birth weight of 2.0 kg (first centile). She had moderate developmental delay, severe speech delay, a high-arched palate and prognathism. An array on blood lymphocytes was performed and identified no abnormalities. Our SNP mosaicism analysis on saliva identified a gain of 12p in an estimated 44% of assayed cells, suggesting tissue-specific mosaicism as the cause. The event was detected also by confirmatory aCGH from saliva, and interphase FISH on buccal DNA of 100 cells identified a triplication of 12p in 12% of cells. Triplications of 12p (tetrasomy 12p) are the cause of the clinical syndrome known as Pallister–Killian mosaic syndrome (34), which is consistent with many of her phenotypic features and the diagnosis was conveyed to the family.

Patient 263654 required 19 days of neonatal intensive care to manage respiratory distress, jaundice and hypoglycaemia. His speech and language were delayed, and an MRI identified inferior vermis hypoplasia. Fragile X testing was normal. Our aCGH was performed on blood and was normal. Our SNP mosaicism analysis identified a 4-Mb duplication in ∼33% of salivary cells. The BAF pattern of the duplication was consistent with a meiotic origin of the duplication in the trisomic cell line. FISH was performed on blood and buccal tissues, and the event was detected in buccal tissue only, in 25 of 50 examined cells. As only interphase FISH was available for buccal tissue, positional information for the additional allele was not possible. The implicated region overlaps most of 16p11.2, a cytogenetic region in which duplications are well known to cause disruption of speech and language development (35), and this event was considered very likely pathogenic.

Patient 259003 had global developmental delay, no speech and generalized hypotonia. Clinical aCGH (6K BAC array) and testing for Angelman syndrome were normal. Our SNP mosaic analysis on salivary cells identified a 5-Mb deletion in 54% of cells at chromosome 22q, from 22q13.31 to 22qter. Array CGH results showed a slight negative deviation in both blood and saliva probe data, but not detected by the aCGH algorithm. FISH on blood lymphocytes identified the event in 43 of 100 of blood cells. This region overlaps with the well-characterized 22q13 Deletion syndrome, also known as Phelan–McDermid syndrome, which has as its main characteristics global developmental delay, absent or severely delayed speech and hypotonia; these manifestations are consistent with child phenotypes (36) and the mosaic event was considered very likely pathogenic.

Patient 260108 had truncus arteriosis, hypertelorism and feeding difficulties at birth. She demonstrated global developmental delay and required nasogastric feeding. An MRI was abnormal and showed possible arterial shunting. Clinical testing for *SALL1*, *SALL4*, *CHD7* and Prader–Willi syndrome were normal. Our aCGH data in blood showed no abnormalities. Our SNP mosaic analysis identified a 14-Mb duplication on chr17 in ∼45% of assayed saliva cells, confirmed by aCGH on saliva (6K BACK array). This mutation appears to be tissue limited. FISH validation was not possible. Mosaic trisomies of chromosome 17 are associated with substantial heart defects, including truncus arteriosus and Tetralogy of Fallot, as well as speech delay (37), consistent with phenotypes in the proband, and considered the likely cause of disorder.

Patient 263708 required neonatal intensive care with nasogastric feeding. At delivery, the placenta was hypertrophic, and numerous haemangiomata were noted. She had macroglossia, macrocephaly and hepatic haemangiomata, as well as episodic hypoglycaemia, oligodontia, esotropia and gynecomastia. The patient had pigmentary mosaicism following Blashko's lines. Clinical karyotype was normal. Beckwith–Wiedemann syndrome was suspected, but clinical testing was negative. Our analysis of SNP data for mosaicism identified genome-wide skews of BAFs, believed to reflect a cell line with unipaternal disomy (Fig. 5C). Some ten or so examples of genome-wide unipaternal disomy have now been reported, with different underlying mechanisms (27). The dominant manifestation of unipaternal disomic mosaicism is Beckwith–Wiedemann disorder, which is consistent with the majority of the phenotypes in this case. In addition, because Beckwith–Wiedeman is associated with increased tumour risk, this diagnosis can help increase surveillance of tumour development through increased screening (38). Given the overlap of phenotypes known in genome-wide paternal UPD and the child's phenotypes, the variant was considered very likely pathogenic.

Patient 258190 required 7 days of neonatal intensive care owing to hypoglycaemia and macrosomia (birth weight and head circumference >99th centile). Congenital muscular torticollis, partial cryptorchidism and vertebral abnormalities (joint fusions in cervical spine) were noted. He had global developmental delay, and autism. Our aCGH assay was performed on blood and was negative, and our mosaic SNP analysis on saliva using MAD was negative. Analysis using triPOD on saliva detected a low-level trisomy on chromosome 20. FISH confirmed trisomy in 30% of cells from buccal sampling but absent in cells from lymphocytes, suggesting the mutation is likely tissue limited. Mosaic trisomy 20 syndrome includes head tilt, developmental delay, autistic features, spinal and genital abnormalities (39), all phenotypes consistent with those observed in this patient; therefore, the mosaic event was considered very likely pathogenic.

Patient 259709 required neonatal intensive care for 31 days with enteral feeding. Developmental milestones were delayed: sitting independently was achieved at 23 months and walking independently began at 3 years. At recruitment, recorded phenotypes included joint laxity, hyperextensible skin, anterior ‘beaking’ of lumbar vertebrae and delayed speech and language development. Our analysis of SNP data identified a chromosome-wide LOH on chromosome 14 in ∼65% of assayed salivary tissue. Informative parental genotypes overlapping the mosaic region identified that the LOH resulted from a mosaic loss of the maternal allele (Supplementary Material, Fig. S5). Loss of heterozygosity may be pathogenic by causing imprinting disorders or by inheritance of a deleterious variant, present from a carrier parent, to homozygosity. Constitutive UPD 14 maternal is known to cause Temple syndrome, for which feeding difficulties at birth, joint laxity and developmental delay are present (40). These features are consistent with the child's phenotypes and considered very likely pathogenic.

Patient 257978 had thoracolumbar scoliosis, seizures, somnolence and abnormality of neuronal migration. She demonstrated profound intellectual disability and achieved no developmental milestones. Clinical karyotyping and telomeric MLPA were normal. Our SNP mosaicism analysis identified an 80-Mb loss-of-heterozygosity region on chromosome 5 in 24% of assayed salivary cells. We suspected that conversion to homozygosity of a deleterious variant in the LOH region may underlie the pathogenicity. To investigate this, from exome sequence data, we inspected rare (<0.5% minor allele frequency) variants that led to missense and loss of function mutations in genes overlapping the LOH region. Of seven such variants, the most interesting candidate was a missense variant in *N4BP3*, a gene recently reported to be required for normal neuronal axonal branching (41). We inspected the sequencing reads of this variant to test whether the deleterious allele was skewed towards homozygosity and found that of the sequencing reads overlapping this variant position, 46 supported the alternate alleles, whereas only 28 supported the reference allele, suggesting that the alternate allele is homozygous in the mosaic cell line. Nevertheless, this gene has not previously been implicated in DD; therefore, a definitive relationship between this variant and the phenotype in the child was difficult to assess, and the variant as considered possibly pathogenic.

Patient 259029 was born at 40-week gestation with a birth weight of 3.3 kg (41st centile). The child has dysmorphic facies including severe hypertelorism, and clinical testing for craniofrontonasal dysplasia was negative. Our aCGH on saliva was not obviously deviated. Our mosaic analysis detected a low-clonality (23%) 3-Mb mosaic event on chromosome 11, with a small elevation of LRR (0.09). Intellectual disability and hypertelorism are shared phenotypes with patient 255428 in the Decipher database with an overlapping duplication. This region contains *ALX4*, a gene implicated in skull ossification defects, which may be consistent with hypertelorism (42). However, this region has not been consistently identified with other specific phenotypic features in the child, and therefore, the variant was considered possibly pathogenic.

## Discussion

The main aim of this study was to investigate whether children with DD have a significant burden of mosaic structural abnormalities relative to age-matched controls. We estimated a ∼40-fold enrichment of mosaicism in cases compared with controls. Using single-sample and trio-based approaches, we calculated that 0.9% of DDD probands had large-scale mosaicism. The substantial burden in cases suggests that many of these events were pathogenic. We assessed whether the phenotypes in each child were consistent with the mosaic mutations and concluded that 10 of 12 were highly likely to be pathogenic.

One component of this study explored the relative performance of single-sample versus trio-based mosaic detection methods. Both methods discovered a majority of the total detections, and neither software tool was clearly advantageous compared with the other. triPOD identified two events of lower clonality not found by MAD. While MAD has diminished sensitivity to low-clonality events, it does not require complete trio data, a resource not always available; in this analysis, two real mosaic events detected by MAD lacked complete trio data and were not analysed by triPOD. Also, one third-haplotype gain was not found by triPOD, and the false-positive rate of triPOD was higher than MAD. These findings suggest that employing either tool can identify the majority of mosaic events but that maximal sensitivity can be gained by leveraging the complementary strategies of both tools if trio data are available.

Assessing the pathogenicity of mosaic copy number and copy neutral events requires several assumptions, primarily, that events present in mosaic form cause phenotypes similar in character, if perhaps less severe, than events present in constitutive form. We used this assumption when assessing pathogenicity of the mosaic events. The majority of events detected were copy number variable mosaicism, which is consistent with previous studies, such as Conlin *et al.* (17). However, in contrast to the study of mosaic aneuploidy, we found much lower levels of sex chromosome aneuploidy (0 in 1303, compared with 9 of 2019), and only a single event in our study was whole chromosome in size. This may be due to differences in ascertainment, as ∼80% of DDD probands were pre-screened by clinical aCGH testing, which would have high sensitivity to detect chromosome-size CNVs present in a majority of cells. In addition, sex chromosomal aneuploidy results in distinctive phenotypes, which are likely trigger extensive genetic investigations; this may compound the bias against recruiting such patients to a research study focusing on undiagnosed patients. For these reasons, our estimate of mosaic frequency in children with undiagnosed disorders is likely an underestimate of frequency among all children with DD.

Mosaic copy number events were typically not detected by standard array CGH analysis. The detection of mosaicism requires both the event to be present in the assayed tissue and sensitive methods that are tailored to identify minimal skews in either intensity, or allele fraction. No large mosaic copy number events were identified in healthy controls, supporting prior evidence that large copy number events are highly pathogenic. On the other hand, one LOH type, a category of mutation imperceptible by aCGH, was detected in healthy controls. While constitutive LOH has been identified in 1–1.5% of children with DD (43,44), a significant burden compared with the population level rate (1 in 3500), the cases studied here did not have a statistically significant enrichment of LOH mosaicism (*P* > 0.05). It remains to be seen whether with increased sample sizes, a burden may become apparent, especially with respect to chromosomes sensitive to imprinting disorders.

The filtering strategy used to identify structural mosaic events was tuned to identify large (2 Mb or larger) mosaicism, a size that allowed fair comparison across data sets given the variability in SNP density. Intuitively, larger events are more likely to be associated with pathogenicity, and empirical observation demonstrates that larger constitutive CNVs are rarely found in healthy children (45). More powerful genetic assays, such as high-depth whole-genome sequencing, will enable a higher-resolution comparison of mosaic events at smaller sizes and allow improved detection of pathogenic mosaicism (2).

The strategy of using inherited duplications to characterize BAF and LRR properties of constitutive duplications for exclusion of putative detections with similar BAF and LRR profiles may have inadvertently filtered some mosaic duplications of very high clonality. As the TEDS data set had SNP data with a higher noise level compared with DDD, this effect may have been more pronounced in the TEDS analysis, which could potentially result in an underestimate of mosaicism in this control group. Nevertheless, the data quality from TEDS was sufficient to detect the size and clonality of mosaic events that were detected in the other cohorts.

The SNP data in the DDD study were derived by salivary DNA extraction. While salivary sampling is non-invasive and represents a mixture of two tissue types (epiderm via buccal tissue epithelium and mesoderm via lymphocytes) (46), this may have limited our sensitivity to low-clonality events confined to a single tissue type. Because ALSPAC and TEDS data were derived from only one tissue type (blood) and the distribution of mosaic events may differ across tissue types, it is possible that our frequency comparison of mosaicism between cases and controls may have been partially confounded by hidden stratification, and indeed some mosaic abnormalities (such as the 12p tetrasomy leading to Pallister–Killian syndrome) are rarely detected in blood; however, the finding that the majority (6 of 8) of mosaic events in saliva were also detected in blood suggests that this effect may be minimal. In addition, this may provide some evidence that mosaicism underlying DDs need not propagate into all germ layers to result in syndromic dysfunction. However, our assessment of tissue distribution was limited, as we did not have access to endoderm-derived tissue, and factors that hinder the extrapolation of germ layer distribution from assayed tissue distribution, such as purifying selection against deleterious mosaicism and sampling error, may have played a role.

Detection of mosaicism in probands and subsequent genetic diagnosis offers reassurances to parents that a subsequent child is not at increased risk of developing the same mutation. Nevertheless, the majority of children with previously undiagnosed genetic disorders still receive no genetic diagnosis after extensive interrogation, including aCGH, exome and SNP-based analyses. Improved detection of all forms of mosaicism is needed, including smaller mosaic abnormalities, such as indels and point mutations. This will require further reductions in sequencing cost and the development of accurate sequence-based mosaicism detection algorithms.

## Materials and Methods

### Description of studies

The DDD Study is a parent-offspring trio study with a main objective of identifying the disease-causing variants in a sampling of 12 000 children with undiagnosed severe developmental conditions. The Scottish Family Health study is also a trio study, designed to study the genetics of complex traits. Both the DDD and SFHS cohorts were processed on the same custom Illumina^®^ SNP genotyping chip, a design combining 733 059 HumanOmniExpress-12v1_A-b37 positions and 94 840 additional selected positions. DNA was sourced from saliva using Oragene^®^ OG-500 (parent) or OG-575 (child) collection tubes (DNA Genotek, Inc.). Genotyping was performed using Illuminus (47), recorded in PLINK format (48) and converted to VCF format (49) using plinkseq version 0.08. Probe-level quality control measures selected polymorphic, well-covered positions that were absent from copy number regions of at least 1% frequency (as calculated from a composite of multiple CNV studies) (50,51). This resulted in 679 891 assayed positions (Supplementary Material, SNP probe selection). Samples were not excluded on outlier levels of BAFs or LRRs because large (especially genome wide) mosaicism will skew these measures and we wanted to prevent unintentional filtering of real mosaicism. The SFHS set is a trio study composed of young adults who lacked delays in development and is considered here as a control study without phenotypic selection.

We collected SNP data from two prospective, longitudinal, birth cohort studies: TEDS and ALSPAC. The child participants from Avon Longitudinal Study of Parents and Children (ALSPAC), a cohort called ‘Children of the 90s’, consists of ∼15 000 children. Illumina SNP genotyping was available for 8970 unique samples. BAF and LRR metrics were derived from raw data using published guidelines (52) (Supplementary Material, : ALSPAC LRR and BAF derivation). For 5667 samples, DNA was sourced from cell line material, 3290 from blood or tissue and 13 had unknown origin. The SNP genotyping chip assayed 478 184 sites on autosomes and chromosome X aligning to GRCh19 and absent from copy number regions of at least 1% frequency. Samples were excluded from selection as controls if the child had phenotypes suggesting developmental problems. Samples were excluded from the control cohort if either of the following phenotype exclusion criteria were met: (sa032a) child has ever had developmental delay: yes; (kd075) [parent] worries over development (kd705) above zero. The ALSPAC study website contains details of all the data that are available through a fully searchable data dictionary: http://www.bris.ac.uk/alspac/researchers/data-access/data-dictionary/. Ethical approval for the study was obtained from the ALSPAC Ethics and Law Committee and the Local Research Ethics Committees.

The TEDS includes ∼13 000 unrelated twin pairs from England and Wales. A main aim of the study is the investigation of genes and environment on cognitive and behavioural development in children. SNP genotype data were derived from buccal swab sampling using Affymetrix 6^®^ chips. This genotyping chip assayed 695 017 sites on autosomes and chromosome X aligning to GRCh19 and absent from common copy number regions (Supplementary Material, SNP Probe Selection). Samples were excluded from selection as controls if the child had phenotypes suggesting perinatal or developmental problems at 4 years were noted: perinatal outlier overall exclusion ‘YES’, medical exclusion ‘YES’, talking problem (dhtalk1) ‘YES’ or above 90th centile for total behaviour problems (dbhbeht1 and dsdbeht1).

### Mosaic event detection and filtering strategies

We implemented two software packages for detection of mosaic events from SNP genotyping data: (1) MAD, which detects a mosaic segment in a single sample as a genomic region with a consistent skew in BAFs (12) and (2) triPOD, which detects a mosaic segment in a proband of a patient–mother–father trio as a genomic region of proband BAFs inconsistent with parental genotypes (13). The advantage of triPOD is an increased sensitivity compared with MAD for detecting events of low clonality; however, triPOD additionally requires parental genotype data, which are not available in all studies.

MAD identifies mosaic segments as clusters of B allele frequencies (BAFs) with a similar skew statistically unlikely to be from constitutive frequencies, where expected BAFs for AA, AB and BB genotypes correspond to 0.0, 0.5 and 1.0 allele frequency. Pertinent attributes of MAD-predicted events include the start and end coordinates, and the average LRR and degree of BAF skew from expectation (‘B-deviation’). Log R Ratio is used to classify the mosaic event by type (loss, gain or loss-of-heterozygosity), whereas B-deviation is useful to assess the proportion of assayed cells with the mosaic event. The following default parameter values were used: aAlpha = 0.8, *T* = 9, MinSegLen = 75. We implemented MAD for all samples from all cohorts.

Initial testing on all 5103 DDD and SFHS samples produced 2299 mosaic putative detections. This is several hundred times higher than the expected number and manual inspection of a large subset identified several sources of technical error, most notably (1) hypersegmentation, (2) segments with skewing of unimodal heterozygous BAFs and (3) segments of constitutive regions of homozygosity and no heterozygous genotypes. We developed a computational strategy to mitigate these errors through automated filtering. First, we managed hypersegmentation by merging nearby (within 1 Mb) segments representing the same event type (loss, gain or LOH) and averaged LRR and B deviation among pre-merged fragments for the merged segment. Segments beyond 2 Mb in size after merging were retained. The next step of the filtering strategy implemented a peak-calling strategy to distinguish between segments with bimodal BAF clusters, segments with skewed unimodal BAF deviations and constitutive regions of homozygosity (Supplementary Material, Computational Filtering). This computational filtering strategy reduced the number of putatively mosaic segments entering manual curation in the combined DDD and SHFS cohorts by ∼90%.

In addition to MAD, triPOD was used to scrutinize detection of low-clonality events. Default settings (alpha = 0.1, nc_thresh = 0.03) were used, except for genome build, which was changed to ‘hg19’. All putative detections of at least 5 Mb were manually reviewed. There were ∼1000 putative detections at 2 Mb, and ∼200 events were reviewed, which identified 2 error modes: no deflection in BAFs (spurious) and CNV present in parent (inherited). Owing to the large number of detections, and our rationale to use triPOD mainly for the detection of low-clonality events, we implemented computational filtering to select segments at least 2 Mb and had median BAFs of <0.70. We also observed several hundred events with BAF values of ‘NA’ or 0.50 (no BAF shift), which appeared spurious, so we implemented a 0.51 minimum threshold cut-off. triPOD identified 11 events with highly skewed BAFs and LRRs that were suggestive of inherited CNVs, and 10 of 11 CNVs were also present in a parent, substantiating the constitutive nature of the event and that the event was inherited and therefore not mosaic, whereas the remaining event clustered with the inherited events was considered likely constitutive.

### Genotypic–phenotypic workup

Each Decipher child enrolled in the DDD study was examined by a clinical geneticist. The patient encounter included detailed family history, prenatal, perinatal and the neonatal period. Assessment of development milestones was performed, and phenotypic features were recorded in Human Phenotype Ontology format (HPO format). In addition, clinical photographs were uploaded with parental consent (24, in review). Mosaic events were assessed for pathogenicity using genomic disorder databases, and exome sequence analysis was reviewed by a multidisciplinary team of molecular scientists and Clinical Geneticists.

The mosaic events of copy number type were assessed for overlap with known genomic disorders. Each region was cross-referenced with Decipher syndromic GRCh37 regions, and OMIM morbid map (http://www.omim.org/). Genomic disorders caused by CNVs of the same direction (losses or gains) were selected. For LOH events, the exomes were interrogated for rare variants genotyped as heterozygous or homozygous in these regions. Exome sequencing was performed as fully described elsewhere (53). In brief, the exome capture design was Agilent^®^ SureSelect v.3 50-Mb baits and augmented with 5 Mb of custom regulatory sequences. Sequencing was performed using Illumina^®^ HiSeq 2000 platform to greater than 50× mean coverage using paired-end 75-bp read-length sequence reads. Alignment to the genome reference GRCh37, version hs37d, used bwa (54) version 0.5.9. Quality control filters (genotype quality <30.0, homopolymer runs >5, variant quality by depth <5.0, read depth <4 or >1200 and strand bias >10.0) were applied.

## Supplementary Material

Supplementary Material is available at *HMG* online.

## Funding

The DDD is supported by the Health Innovation Challenge Fund (HICF-1009-003), a parallel funding partnership between the Wellcome Trust and the Department of Health, and the Wellcome Trust Sanger Institute (WT098051). The Twins Early Development Study is supported by the UK Medical Research Council (G0901245; and previously G0500079). The ALSPAC study is supported by the UK Medical Research Council, and the Wellcome Trust (Grant: 102215/2/13/2). The University of Bristol provide core support for ALSPAC. N.J.U., H.S. and T.G. work within the MRC Integrative Epidemiology Unit supported by Medical Research Council (MC_UU_12013/1-9). Generation Scotland: Scottish Family Healthy Study (GS:SFHS) has received core funding from the Chief Scientist Office of the Scottish Government Health Directorates CZD/16/6 and the Scottish Funding Council (Grant HR03006). Funding to pay the Open Access publication charges for this article was provided by The Wellcome Trust.

## Supplementary Material

Supplementary Data
