# Supplementary material for: Mosaic structural variation in children with developmental disorders
Source: Hum Mol Genet. 2015 Jan 29;24(10):2733–45. doi: 10.1093/hmg/ddv033 (PMC4406290; doi:10.1093/hmg/ddv033)
Supplement: Supplementary Data [file supp_24_10_2733__index.html]

Mosaic structural variation in children with developmental disorders — Mosaic structural variation in children with developmental disorders — Supplementary Data 

# Mosaic structural variation in children with developmental disorders

## Supplementary Data

Supplementary Data

**Files in this Data Supplement:**

- Supplementary Data - Docx file
