# Supplementary material for: Mosaic structural variation in children with developmental disorders
Source: Hum Mol Genet. 2015 Jan 29;24(10):2733–45. doi: 10.1093/hmg/ddv033 (PMC4406290; doi:10.1093/hmg/ddv033)

DDD & SFHS Constitutive CNV Filtering

We observed ten putative mosaic detections among DDD and SFHS samples that were suspicious for being constitutive because of the magnitude of upward deviation of LRRs and wide separation of BAFs. These putative events form a cluster at the right extent of the blue (duplications) line (Supplementary Picture 1).


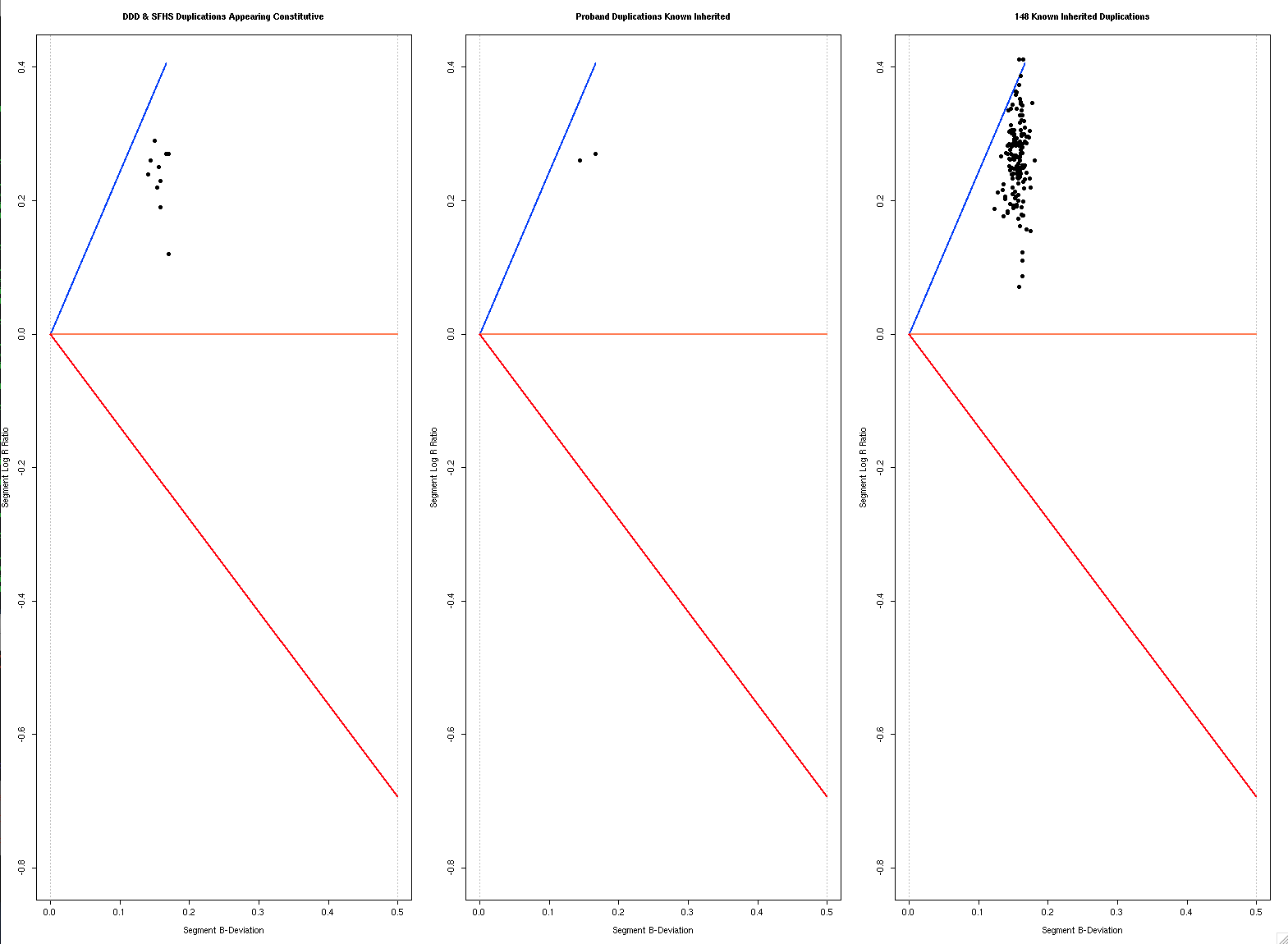


1. Supplemenatary Picture 1

Two of these events were found in probands and parental data were available that showed the same CNV present in at least one parent, substantiating the constitutive nature of these proband events.

Since the DDD and SFHS are trio studies, we selected a list of 1,813 CNVs detected by array CGH and classified as inherited CNVs (Fitzgerald 2014, submitted), and manually curated this list to 148 high quality set of inherited CNVs at least 200 kb in size. We calculated the B-allele deviation (B-dev) and LRR for each CNV and plotted the results, observing that all ten suspicious duplications overlapped with the ‘cloud’ of inherited duplications, and thus were all very likely constitutive. The dots form a vertical pattern partly because the variance of LRR is higher than the variance of B allele frequencies. The single event (arrow) below the main cluster is the paternal counterpart of a proband duplication (Supplementary Picture 2).


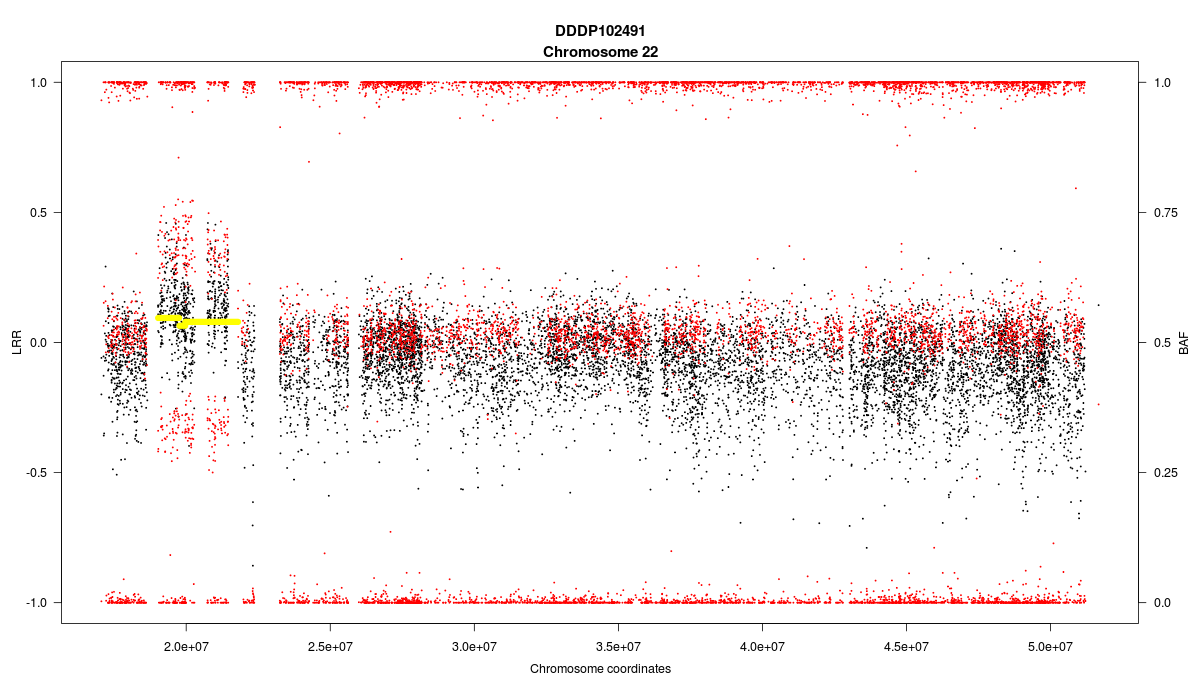

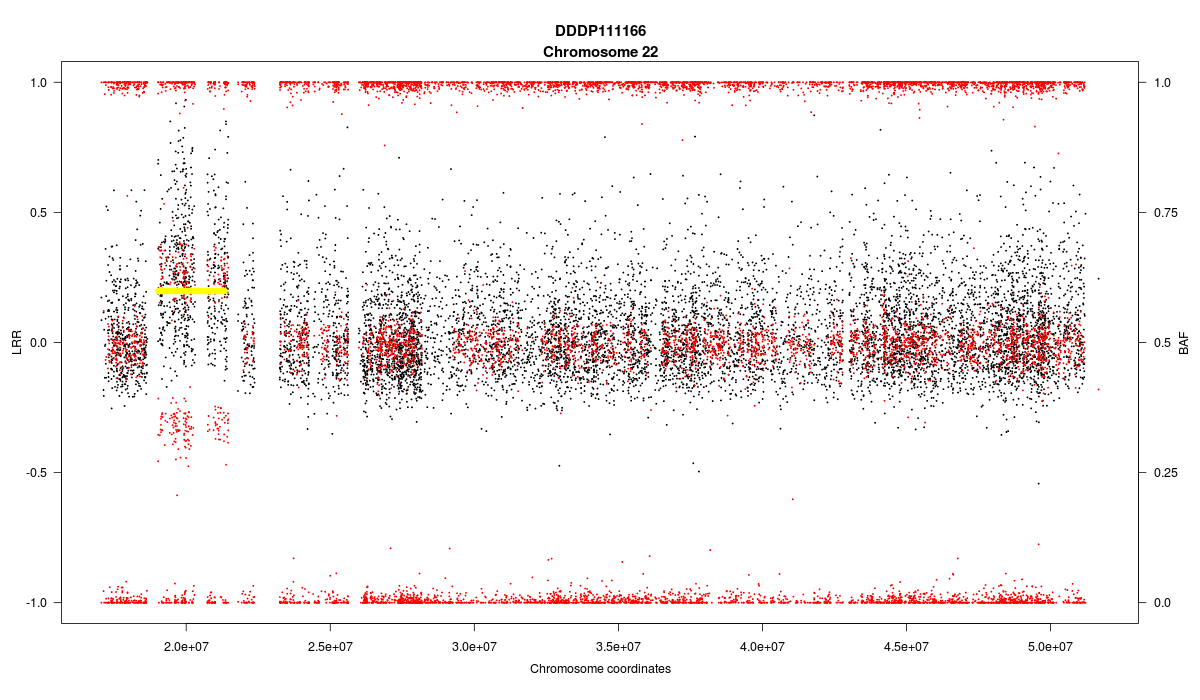


Proband

Father

Supplemenatary Picture 2

Computational Filtration

The merging strategy used to mitigate over-segmentation required manual adjustment on the rare occasion that small fragments were greater than 1 Mb in distance from the next fragment, or when small fragments were misclassified by type and were not automatically merged. For example, one event on chromosome 3 was detected in 23 segments (yellow lines), which could be merged to 6 larger fragments; two of these were classified by MAD as loss of heterozygosity, and four as duplications. This represented over-segmentation of a single mosaic duplication event, and the LOH events were reclassified as duplications, and the entire event merged into one. Another example was a chromosome 14 event, where three small spurious segments of LOH are called within the much larger mosaic duplication. In this case, these three small segments were ignored, and the larger segments representing the real duplication were merged (Supplementary Picture 3)


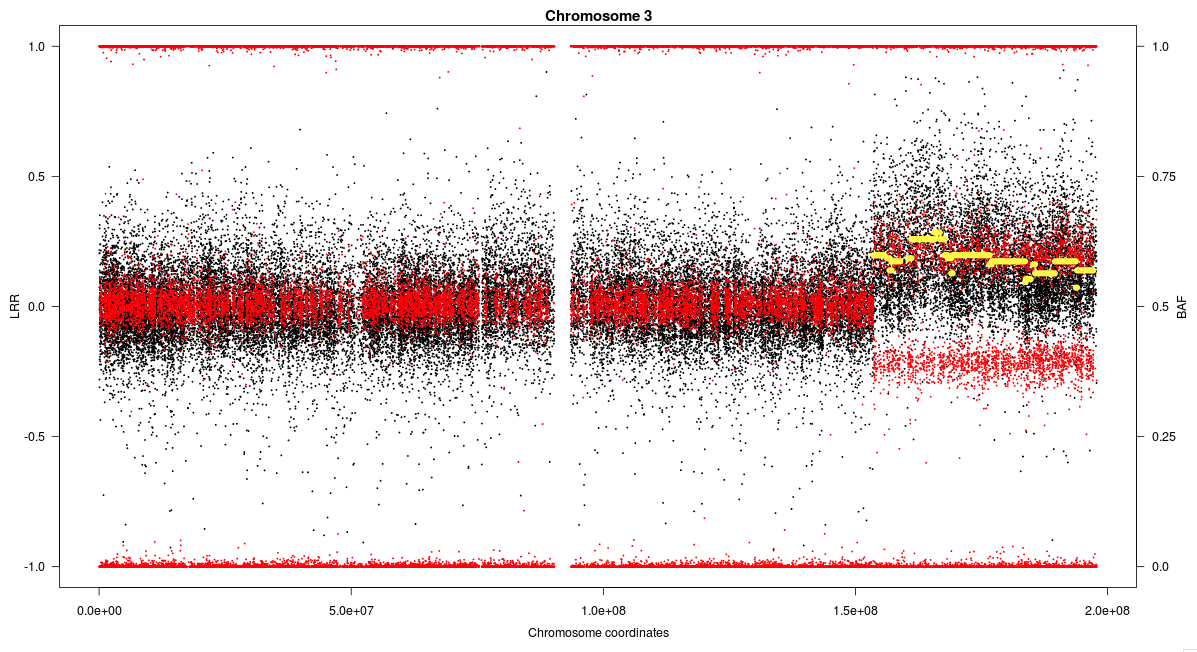

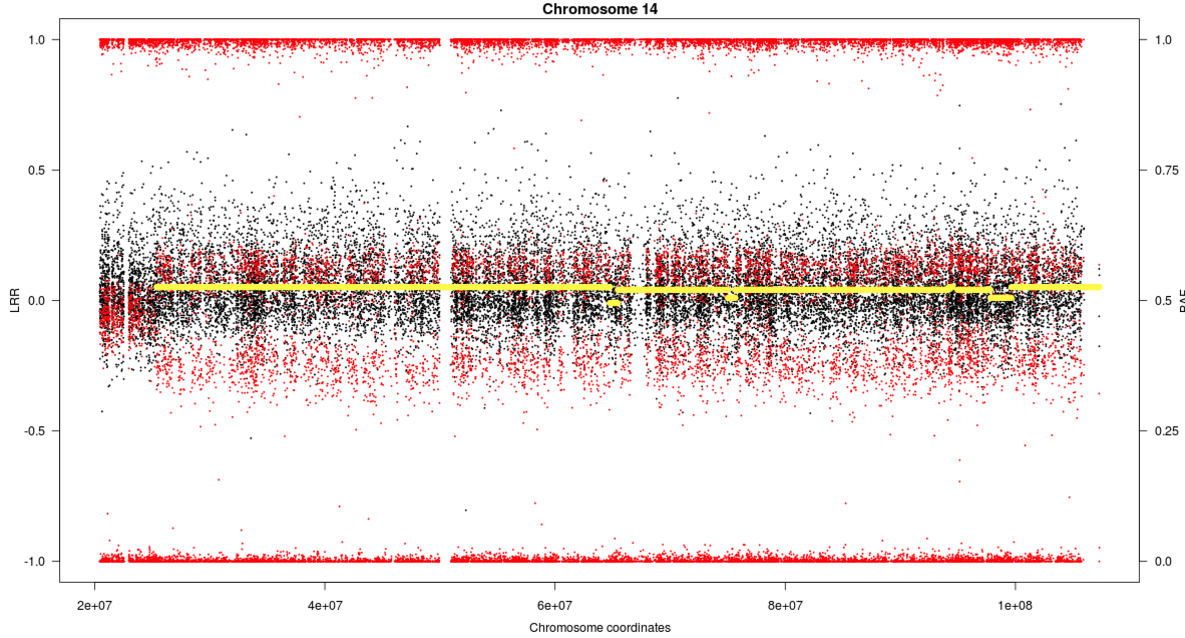


Supplemenatary Picture 3

We developed a peak calling strategy using the R density function. There were hundreds of putative detections found to be subtle unimodal deviations of heterozygous BAFs from an expected BAF of 0.5. Since true mosaic detections result in bimodal deviations from 0.5, we filtered fragments that resulted in unimodal density peaks from those with bimodal density peaks. Specifically, we calculated the peak heights of all heterozygous BAF clusters and found that two metrics, a great relative difference (greater than 5:1) of tallest peak to next-tallest peak was efficient at differentiating unimodal from bimodal peaks, and inspection of segments underlying each category demonstrated that this was effective. Secondly, additional hundreds of putative detections appeared to be small blocks of constitutive homozygosity. Thus, for every segment we calculated the ratio of heterozygous to homozygous SNPs and found that events underlying constitutive homozygosity clustered with a very low ratio of het:hom genotypes; events with fewer than 10% heterozygous genotypes clustered together, reflecting constitutive homozygosity. All segments on chromosome X were manually reviewed, to prevent exclusion of segments in males with aberrant BAF characteristics due to mosaicism in the context of hemizygosity. Examples of segments failing the bimodal density peak filter and het:hom ratio filter are provided (Supplementary Picture 4).


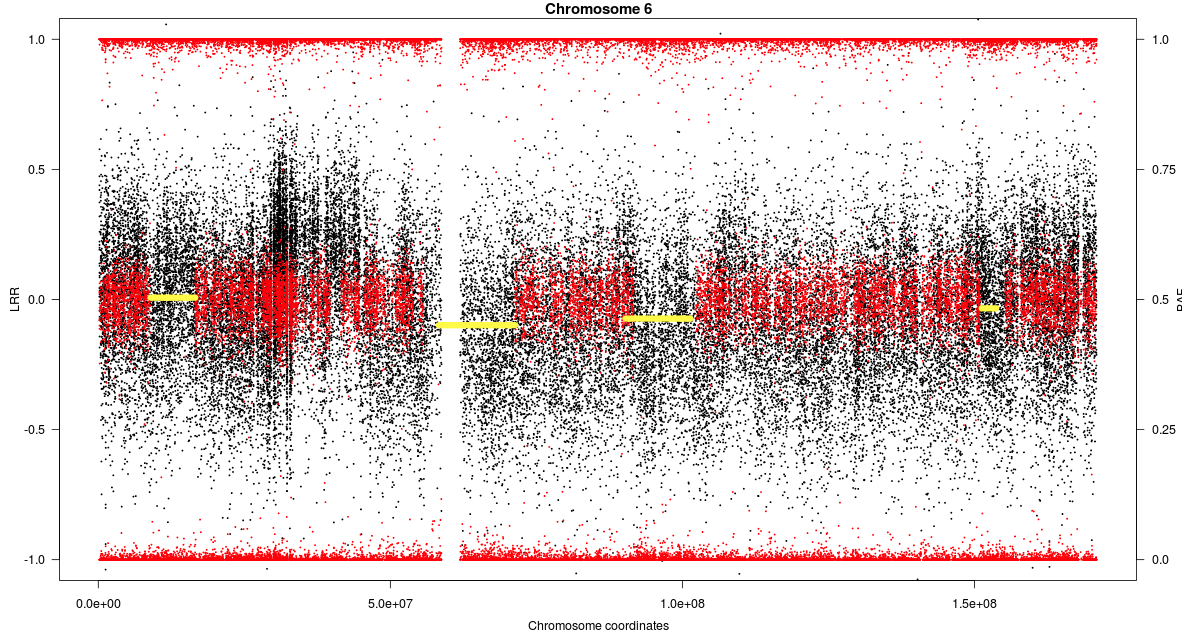

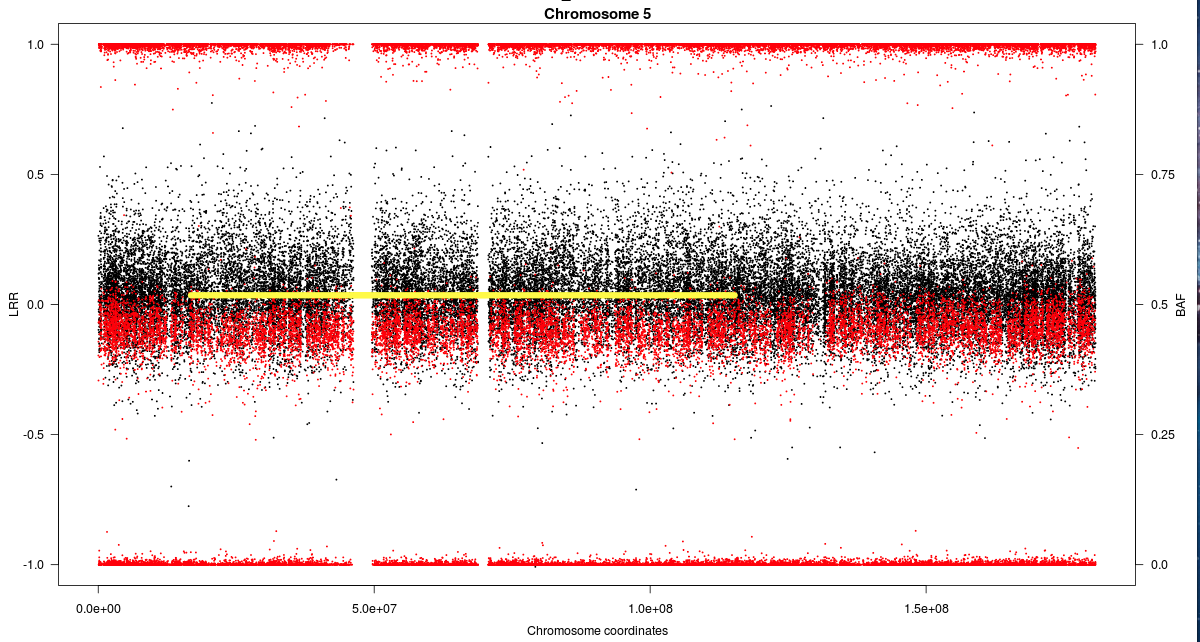


Supplemenatary Picture 4

The computational filtering strategy reduced the number of putative mosaic segments among DDD and SFHS studies from about 2,000 to 81. The majority of the remaining putative events were spurious deviations, including X chromosome events, events in a particularly troublesome region at the centromere of chromosome 11, and small events confined to the peri-centromeric region. There were ten events that were duplication events with LRR and BAF values that were substantially skewed and the possibility that these events were constitutive was investigated (see DDD & SFHS Constitutive CNV Filtering) (Supplementary Picture 5)


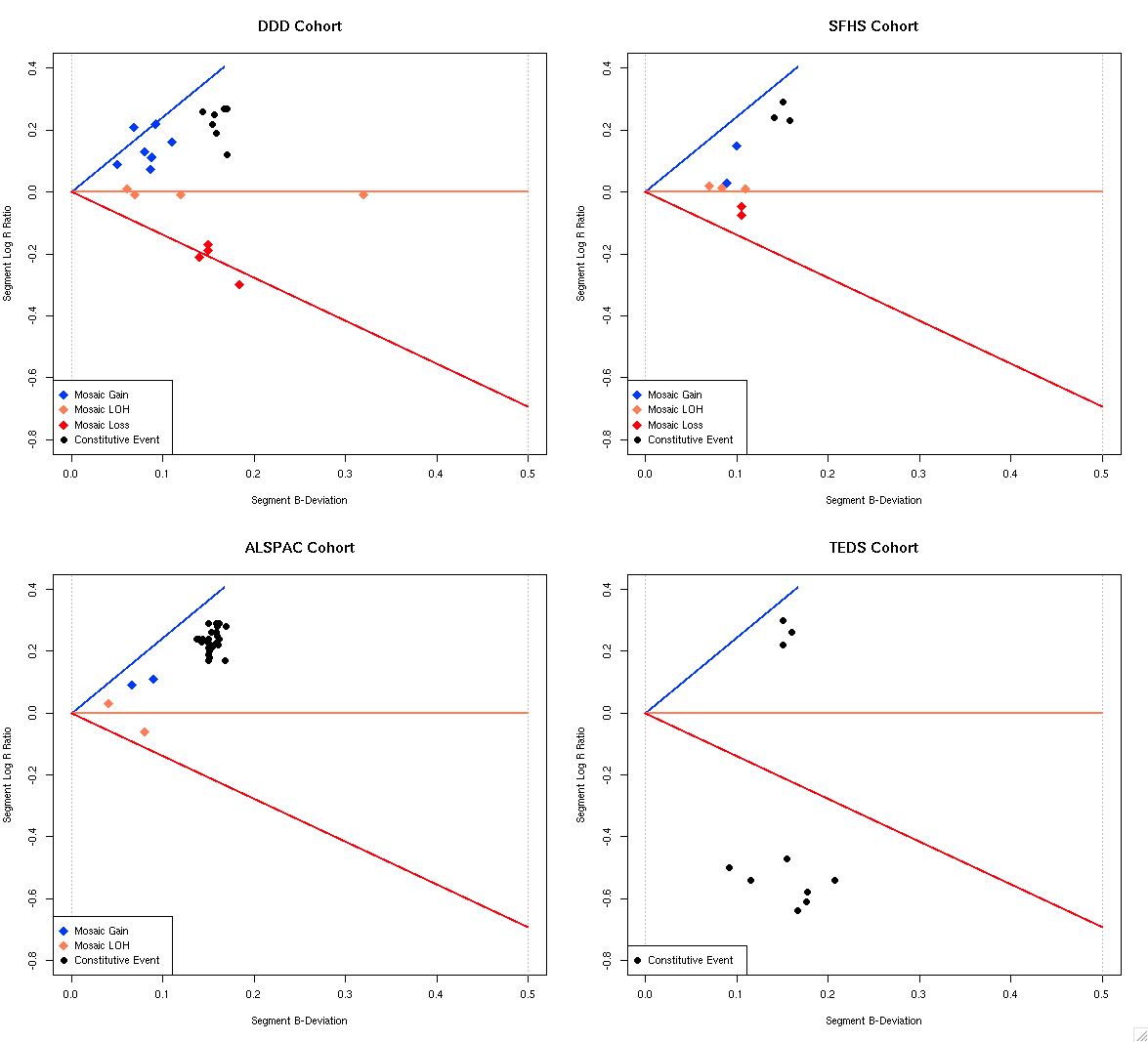


Supplemenatary Picture 5

Small detections confined to pericentromic regions and appearing constitutive were not included in analysis.

The MAD results for the TEDS cohort were merged and filtered as above, and events of 2 Mb size or greater in samples passing phenotypic exclusion criteria were included for analysis. There were 87 putative events at this size or greater; these included 7 events with large skews in LRRs and BAFs, 30 that reflected two sibling contamination events, and the remaining were due to spurious X chromosome deviations in males, and small peri-centromeric events. Four of seven events were deletion events, with BAFs not strictly at 0 and 1, but skewed inwards. These events had consistent levels of LRR and BAFs and clustered together, suggesting they were constitutive events, but skewed due to a noisy background. The remaining three of the seven were gains, and surprisingly, two of these three represented trisomy chromosome X. Extended phenotypic data of these two individuals, including school maths, reading and anxiety levels were scrutinised, but neither child was an outlier in any of these measurements, suggesting their trisomy X was benign or subclinical. The curated mosaic and constitutive segments from MAD analysis for all SNP-based cohorts are provided below. ff

triPOD detection in SFHS yielded 26 putative events, of which 3 were constitutive and 23 were spurious, all but two in a narrow pericentromeric region of chromosome 11.

ALSPAC LRR and BAF Derivation

*Generation of LRR and BAF*

IDAT files and computed LRR and BAF were not available for ALSPAC. Normalised probe intensities were used to estimate LRR and BAF using the approach described in Pfeiffer et al. Reference genotype clusters were derived from the whole cohort (a representative population sample). LRR was computed as the ratio of normalised intensity of a sample to expected intensity (based on linear interpolation of reference genotype cluster intensities for that allelic ratio). BAF was computed as relative position of sample allelic ratio between homozygote (allele dose 0.0/1.0) and heterozygote (allele dose 0.5) reference clusters.

SNP Probe Selection

| DDD & SFHS SNP Probe Quality Control | | |
| --- | --- | --- |
| #Positions | Filtering Step | |
| 810110 | all designed positions | |
| 793968 | removing non-SNV or non [ATCG] positions | |
| 695516 | removing maf < 0.01, hwe > 0.001, missingness > 0.1 | |
| 679891 | removing positions in common CNV regions | |
|  | |  |
| ALSPAC SNP Probe Quality Control | | |
| # Positions | Filtering Step | |
| 610259 | provided QC polymorphic hg18 positions | |
| 500527 | Passed ALSPAC QC | |
| 488199 | Mapping to GRCh37 | |
| 478164 | Outside common CNVs | |
|  |  | |
| TEDS SNP Probe Quality Control | | |
| # Positions | Filtering Step | |
| 723257 | provided QC polymorphic hg18 positions | |
| 710992 | Mapping to GRCh37 | |
| 695017 | Outside common CNVs | |


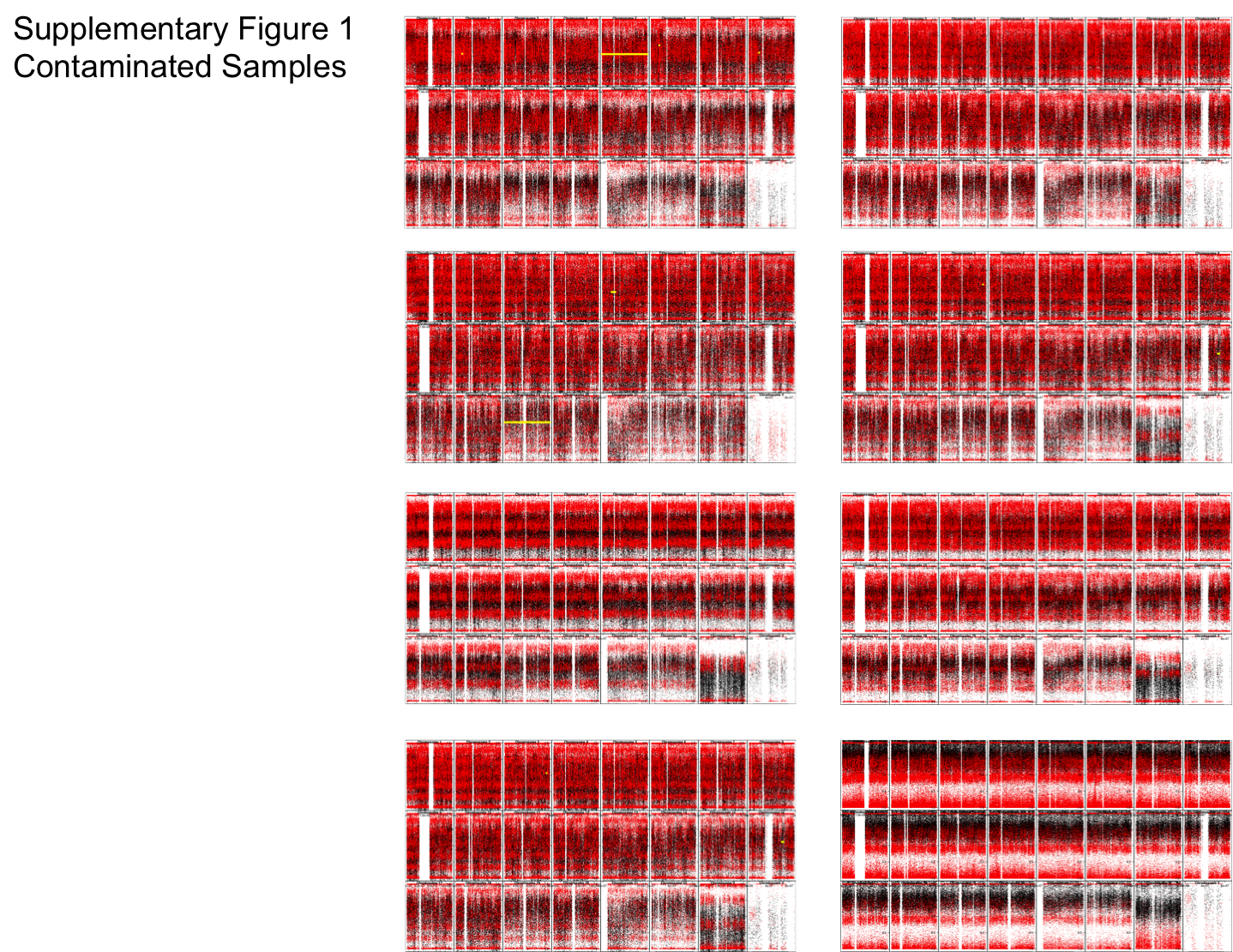


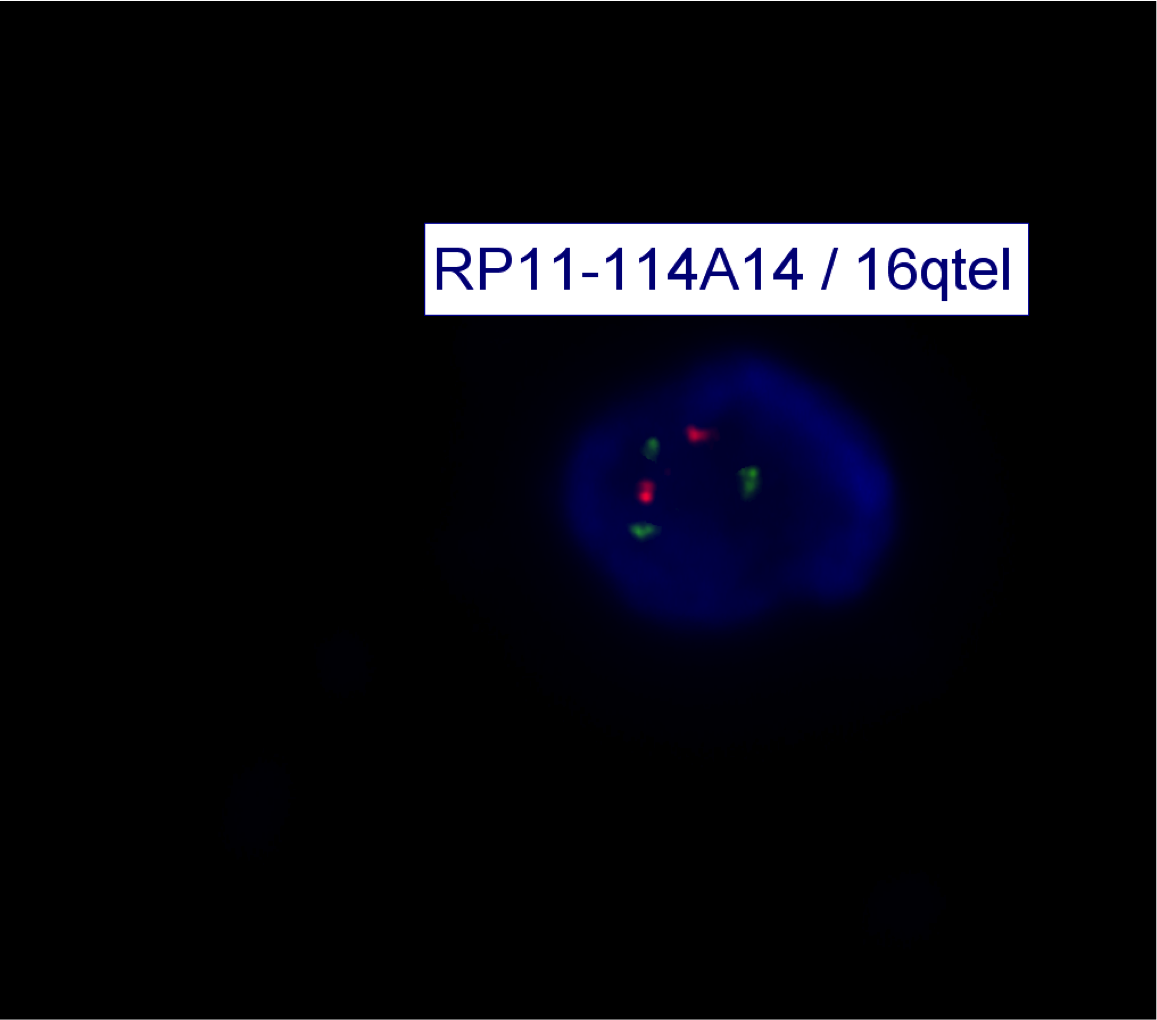


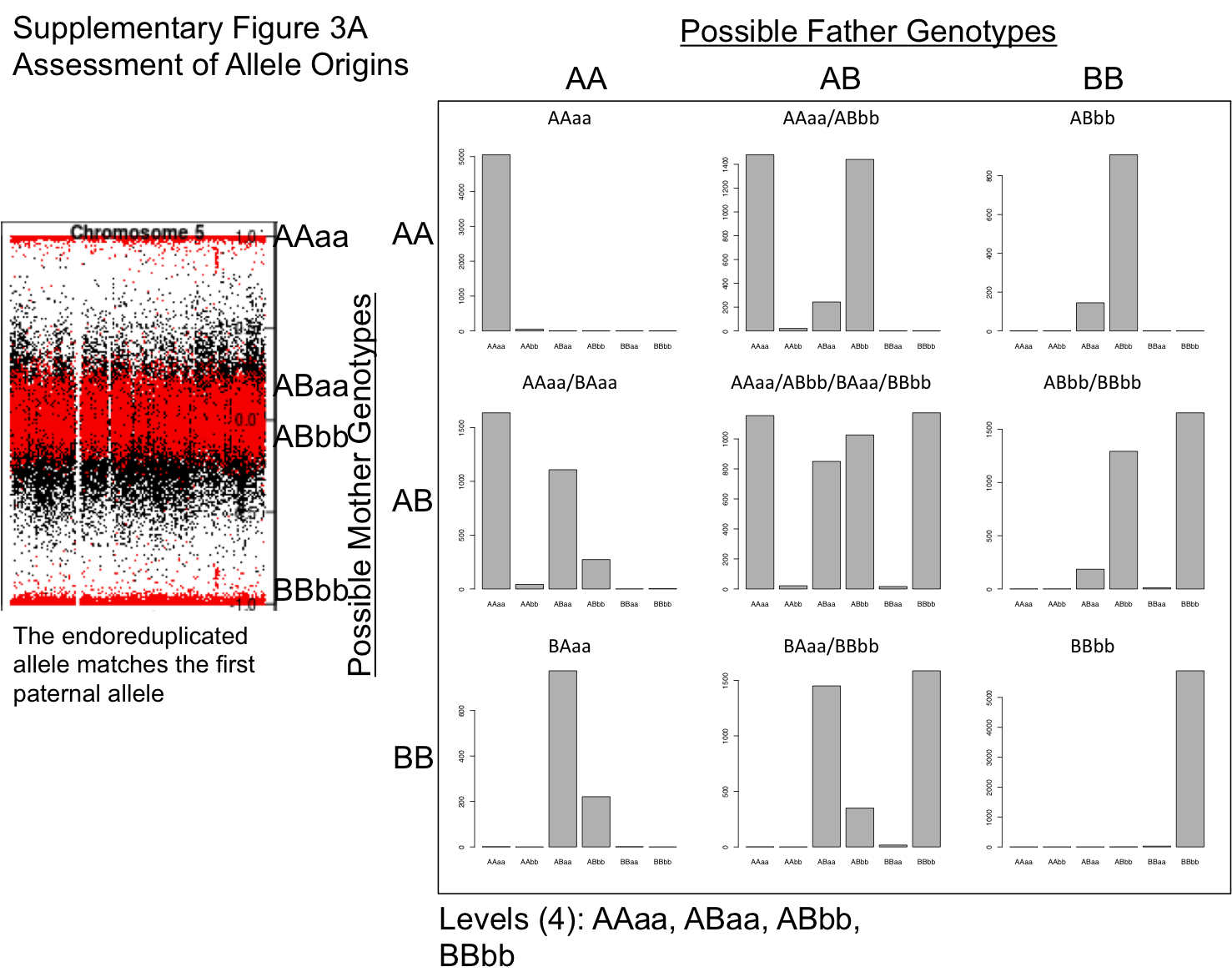


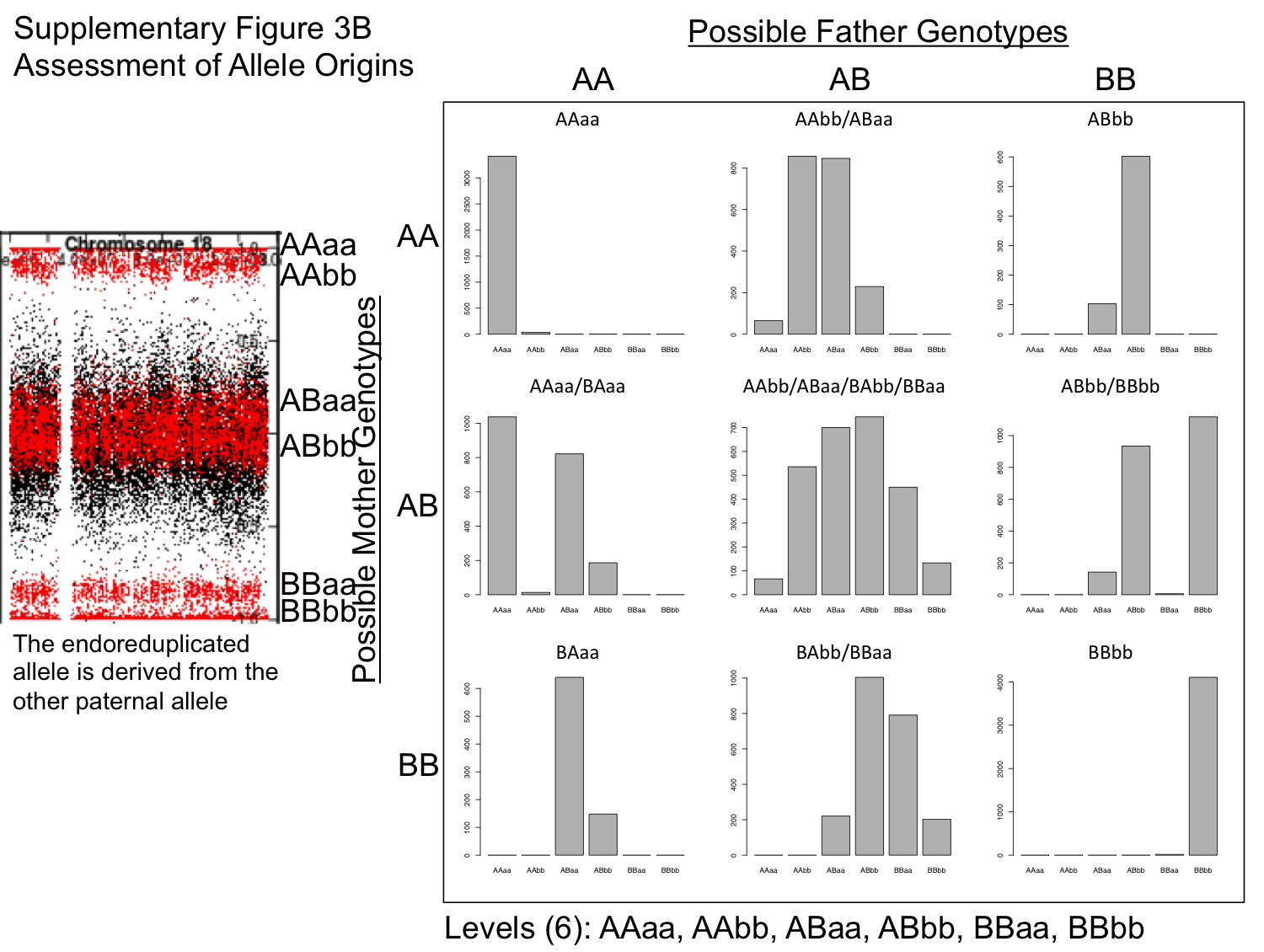


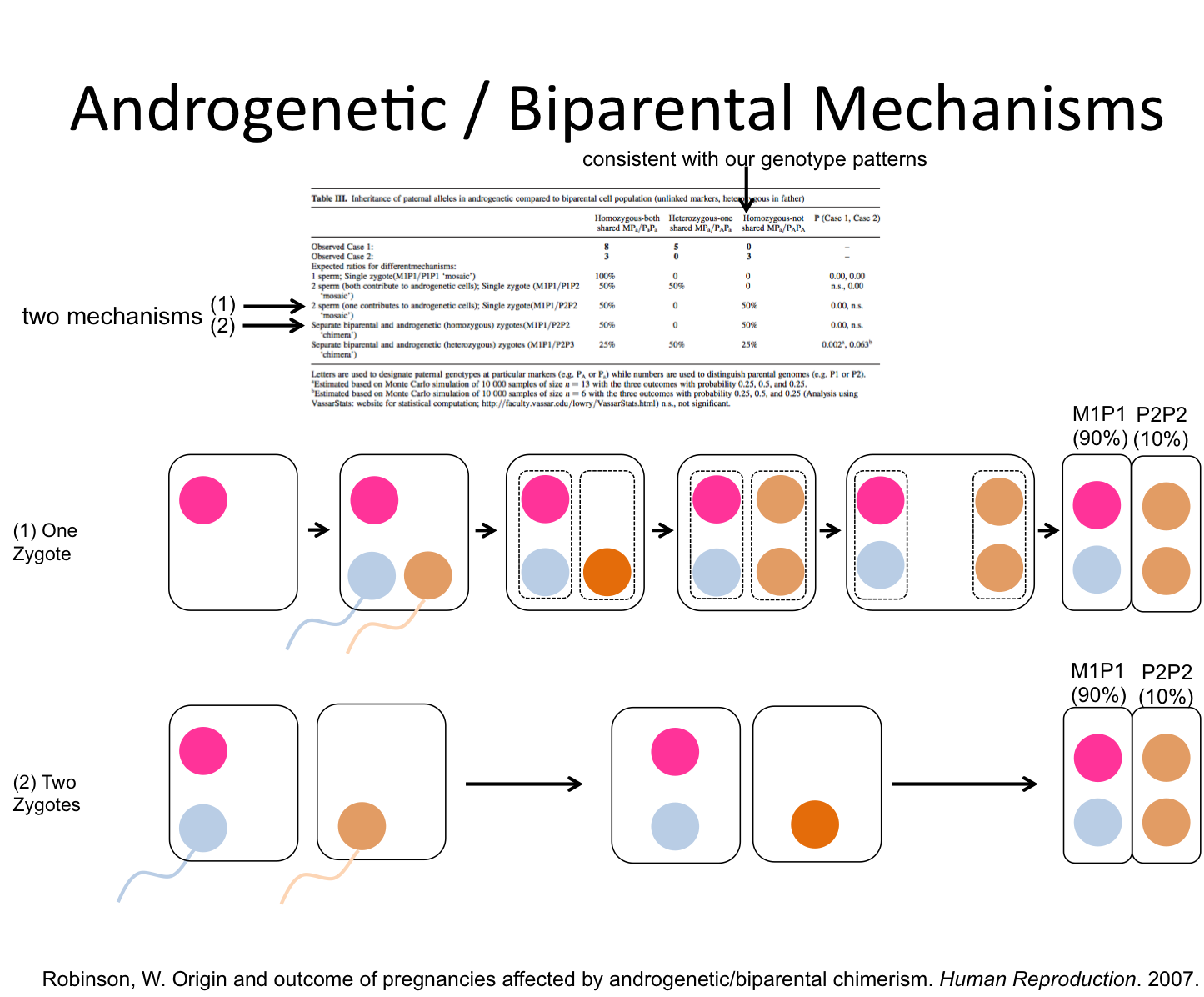


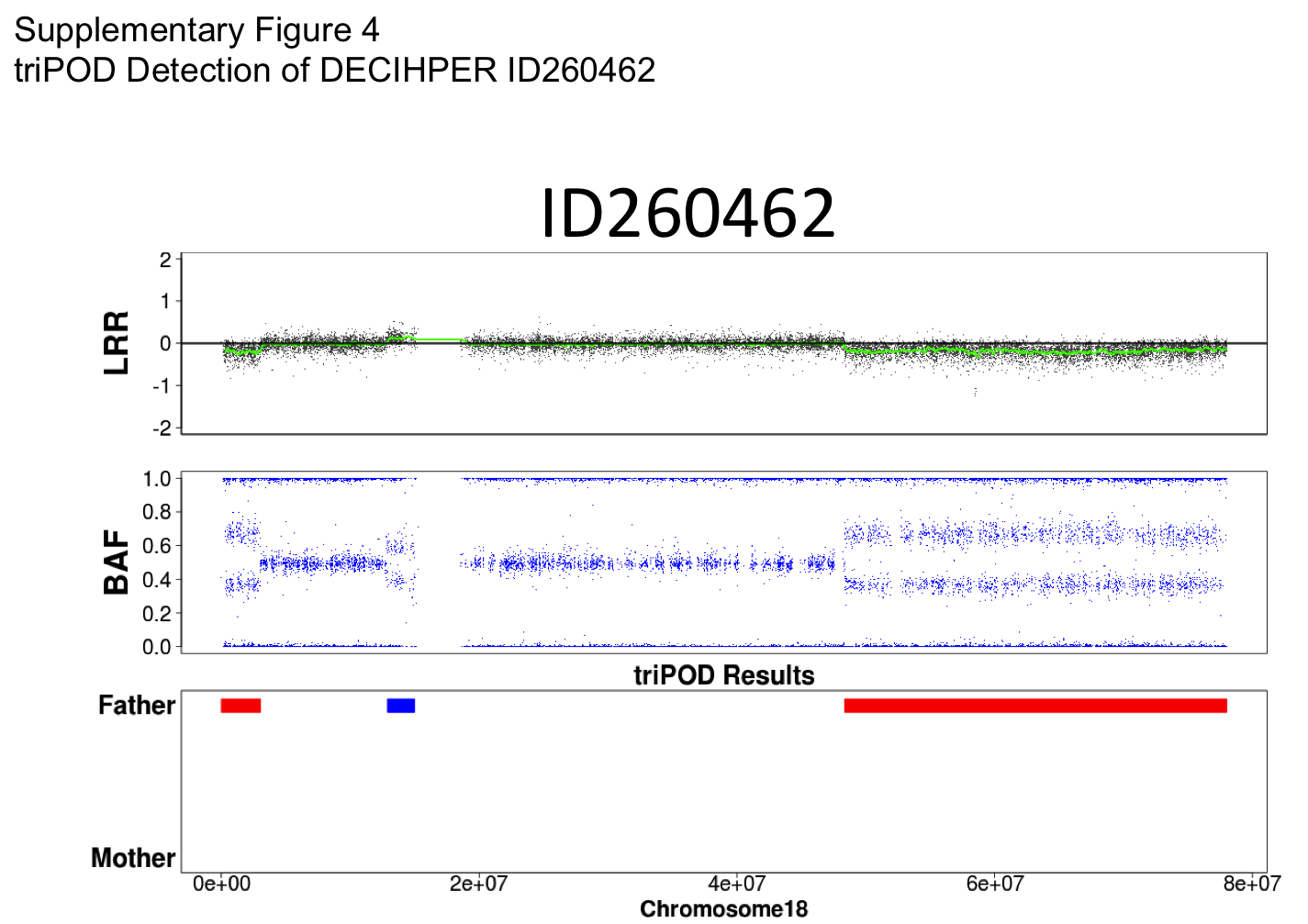


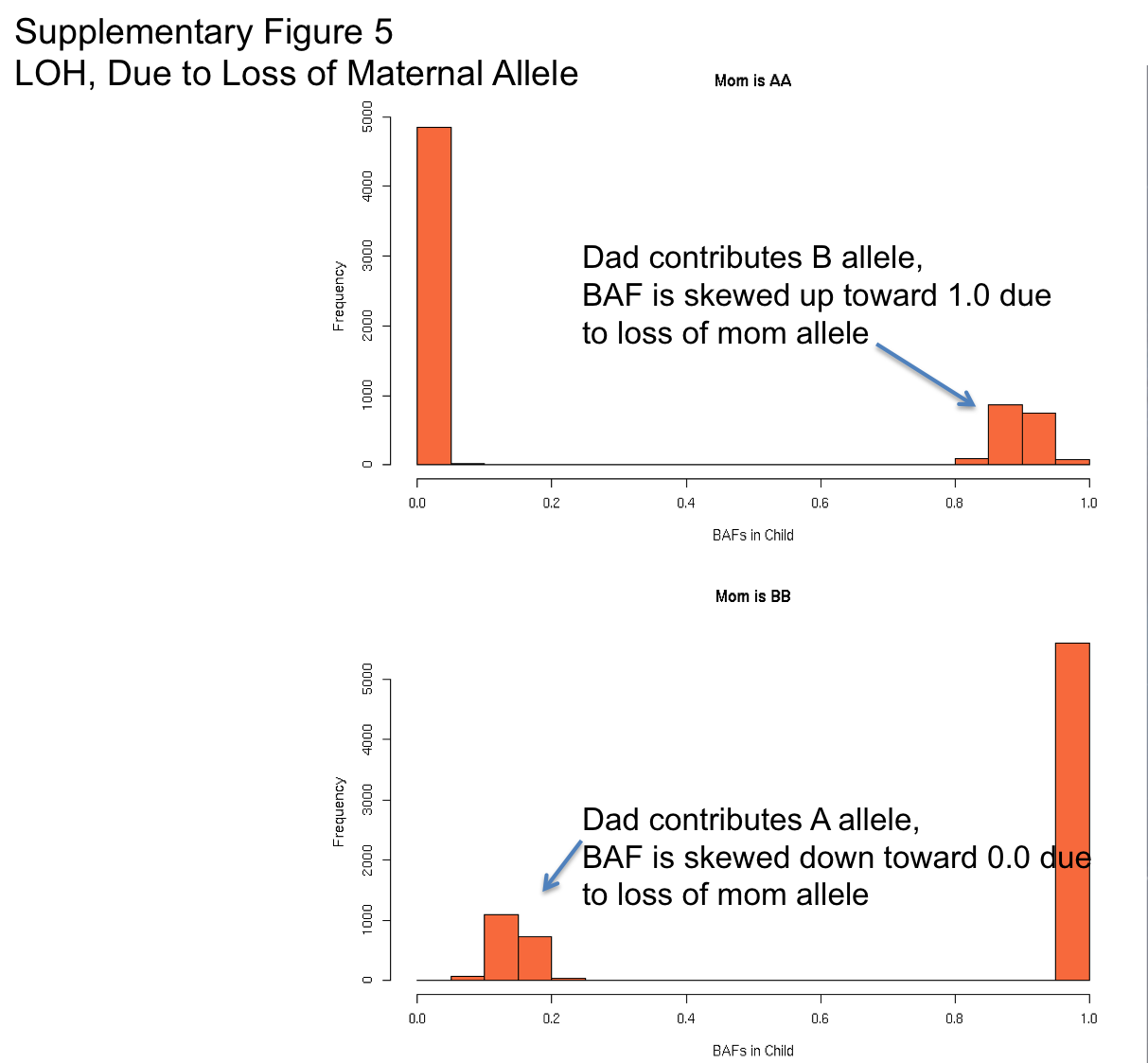

Supplement: Supplementary Data [file supp_ddv033_ddv033supp.docx]
